# Efficacy and safety of Shen Gui capsules for chronic heart failure: a systematic review and meta-analysis

**DOI:** 10.3389/fphar.2024.1347828

**Published:** 2024-04-10

**Authors:** Jiaqi Yan, Chaorong Zhang, Yuanping Wang, Xia Yan, Lili Jin

**Affiliations:** ^1^ Second Clinical Medical College, Guangzhou University of Chinese Medicine, Guangzhou, China; ^2^ Guangdong Provincial Key Laboratory of Research and Development in Traditional Chinese Medicine, Cardiovascular Department, The Fifth Clinical Medical College of Guangzhou University of Chinese Medicine, Guangzhou, China; ^3^ Medical Examination Center, Guangdong Provincial Hospital of Chinese Medicine, The Second Affiliated Hospital, Guangzhou University of Chinese Medicine, Guangzhou, China

**Keywords:** Shen Gui capsule, chronic heart failure, meta-analysis, efficacy, safety

## Abstract

**Background:**

Although Shen Gui capsules (SGCP) are widely used as an adjuvant treatment for chronic heart failure (CHF), their clinical efficacy and safety remain controversial.

**Purpose:**

To assess the efficacy and safety of SGCP in the treatment of CHF through a systematic review and meta-analysis, to provide high-quality evidence for evidence-based medicine.

**Methods:**

Seven databases were searched for randomized controlled trials (RCTs) assessing SGCP for CHF, from inception to 9 January 2023. RCT quality of evidence was evaluated using the Cochrane Handbook for the Evaluation of Intervention Systems to assess risk of bias and Grading of Recommendations Assessment, Development, and Evaluation. A meta-analysis with subgroup and sensitivity analyses was performed using Review Manager 5.4 and Stata 12.

**Results:**

Nine RCTs representing 888 patients with CHF were included in the review. Meta-analysis revealed that SGCP combined with conventional heart failure therapy is more advantageous for improving left ventricular ejection fraction [LVEF; mean difference (MD) = 5.26, 95% confidence interval (CI) (3.78, 6.74), *p* < 0.0000] and increasing effective rate [relative risk (RR) = 1.21, 95%CI (1.14, 1.29), *p* < 0.001] compared with conventional therapy alone. The experimental treatment also reduced brain natriuretic peptide [MD = −100.15, 95%CI (−157.83, −42.47), *p* = 0.0007], left ventricular end-diastolic diameter [MD = −1.93, 95%CI (−3.22, −0.64), *p* = 0.003], and hypersensitive C-reactive protein [MD = −2.70, 95%CI (−3.12,−2.28), *p* < 0.001] compared with the control group. However, there was not a statistically significant difference in tumor necrosis factor-α [MD = −14.16, 95%CI (−34.04, 5.73), *p* = 0.16] or left ventricular end-systolic diameter [MD = −1.56, 95%CI (−3.13, 0.01), *p* = 0.05]. Nor was there a statistically significant between-groups difference in incidence of adverse events (*p* > 0.05).

**Conclusion:**

SGCP combined with conventional heart failure therapy can improve LVEF and increase the effective rate to safely treat patients with CHF. However, further high-quality studies are needed to confirm these findings, due to the overall low quality of evidence in this literature.

**Clinical Trial Registration:** https://www.crd.york.ac.uk/PROSPERO/logout.php, PROSPERO [CRD42023390409].

## 1 Introduction

Chronic heart failure (CHF), an end-stage state of cardiovascular disease, is characterized by stagnation of the pulmonary or somatic circulation and insufficient systemic blood perfusion (McDonagh et al., 2023). Epidemiologic data indicate that 64.3 million people worldwide suffer from heart failure (HF) (Savarese et al., 2023). Coronary artery disease, hypertension, diabetes mellitus, and obesity are the main causes of CHF(Heidenreich et al., 2022; Barghash, 2023); consequently, the average age of patients with HF is decreasing because the prevalence of these diseases continues to rise among young people (Lecoeur et al., 2023). HF also imposes an enormous economic burden on global public health. A study across 197 countries showed that the overall global economic cost of HF in 2012 was approximately $108 billion (Kapelios et al., 2023).

Most importantly, CHF is the leading cause of death from cardiovascular disease (Bozkurt et al., 2023). Thus, HF treatment is aimed at delaying its development, improving cardiac function, relieving clinical symptoms, and reducing mortality. Typically, diuretics, angiotensin-converting enzyme inhibitors (ACEIs), angiotensin AT-1 receptor blockers (ARBs), and beta-adrenergic blocking agents are effective CHF treatments (Heidenreich et al., 2022). Yet despite the maturity of current CHF treatment options, the mortality rate from this condition remains high. A longitudinal analysis of 86,000 patients with HF showed a one-year mortality rate of up to 32% after an HF event (Conrad et al., 2019). The search for safer, more effective CHF therapeutic options thus remains a major challenge. Traditional Chinese medicine (TCM) has recently been found to have significant advantages in the management of chronic diseases, including CHF.

Sen Gui capsules (SGCP) are a TCM preparation approved by the National Medical Products Administration of China in 2019 (Approval number: Z20000060, Specification: 0.3 g/capsule, Shanghai Yudan Pharmaceutical Co.). Animal experiments (Zongduo and Runtang, 2003) have shown that SGCP can dilate the rat coronary artery to increase blood flow, lower blood pressure, increase cardiac output, and protect cardiomyocytes. Clinical trials (Songlin et al., 2022) have also demonstrated that SGCP improves cardiac function, delays HF progression, and is efficacious in the adjuvant treatment of CHF. Although its adjunctive efficacy has been demonstrated, the quality of evidence from these clinical studies has not yet been assessed. To date, there have been no systematic reviews or meta-analyses of the efficacy and safety of SGCP in CHF treatment. Therefore, this study assessed the efficacy and safety of SGCP in the treatment of CHF through a systematic review and meta-analysis, to provide high-quality evidence for evidence-based medicine.

## 2 Materials and methods

This systematic review and meta-analysis was conducted according to the Preferred Reporting Items for Systematic Reviews and Meta-Analyses (PRISMA) (Page et al., 2021) and Cochrane Handbook 2019 (Cumpston et al., 2019) for Systematic Reviews. The PRISMA2020 checklist is detailed in the Supplementary Material S5. The study protocol was registered in the International Prospective Register of Systematic Reviews (https://www.crd.york.ac.uk/PROSPERO/logout.php, PROSPERO, No. CRD42023390409) and had no amendments to the information provided at registration.

To ensure study accuracy, these analyses adopted the consensus statement on the Phytochemical Characterisation of Medicinal Plant extract (ConPhyMP) as a reference when reporting SGCP. We also followed the guidelines for standardizing the scientific nomenclature of botanical drug components. Moreover, we validated these names by cross-referencing them with the websites “Plant of the World Online” (http://www.plantsoftheworldonline.org) and “The World Flora Online” (http://www.worldfloraonline.org) (see compositions of included SGCP in the Supplementary Material S1–S4).

### 2.1 Inclusion and exclusion criteria

#### 2.1.1 Studies

All randomized controlled trials (RCTs) on SGCP for CHF treatment published in China and abroad, in Chinese or English, were included.

#### 2.1.2 Study participants

Patients with CHF who were older than age 18 years, regardless of gender, race, disease duration, or comorbidities, who met the criteria for CHF diagnosis in the 2022 guidelines (Heidenreich et al., 2022) issued by the American Heart Association with New York Heart Association (NYHA) cardiac function classification (Greene et al., 2021) stage II–IV were included.

#### 2.1.3 Intervention

According to the guideline criteria (Heidenreich et al., 2022) for HF management issued by the American Heart Association in 2022, the control group was treated with conventional HF therapy including diuretics, β-receptor antagonists, ACEIs, ARBs, angiotensin receptor-neprilysin inhibitors, or other recommended medications. The experimental group was administered SGCP in combination with conventional HF therapy, with unlimited duration of treatment and medication dosage.

#### 2.1.4 Outcome measures

The primary outcomes were left ventricular ejection fraction (LVEF) and effective rate. Secondary outcomes included hypersensitive C-reactive protein (hs-CRP), tumor necrosis factor-α (TNF-α), left ventricular end-systolic diameter (LVESD), left ventricular end-diastolic diameter (LVEDD), brain natriuretic peptide (BNP), and adverse events. The effective criteria were: NYHA classification improved by ≥ 1 grade, improved HF symptoms and signs, or reduced TCM evidence points by ≥ 30% after treatment. The ineffective criteria were: no improvement or aggravation of NYHA classification or HF symptoms and signs, or reduction of TCM evidence points by <30% after treatment. The treatment effective rate = number of effective treatments/total number of treatments × 100%.

#### 2.1.5 Exclusion criteria

RCTs which included patients with CHF due to congenital heart disease, had incomplete study descriptions, were duplicate studies, or included academic misconduct such as data falsification were excluded.

### 2.2 Search strategy

Seven online databases were searched: China National Knowledge Infrastructure (CNKI); Wan Fang; China Science and Technology Journal Database (VIP); China Biology Medicine disc (CBM); PubMed; Embase; and Cochrane Library. Each was searched for relevant literature on SGCP for CHF from inception until 9 January 2023. The main search terms used in the Chinese databases were “Xinlishuaijie”, “Xinshuai”, “Xingongnengbuquan”, and “Shenguijiaonang”. Those used in the English database included “heart failure” and “shengui capsule”. The specific PubMed search strategy was: (“Heart Failure” [Mesh]) OR (Cardiac Failure)) OR (Heart Decompensation)) OR (Decompensation, Heart)) OR (Heart Failure, Right-Sided)) OR (Heart Failure, Right Sided)) OR (Right-Sided Heart Failure)) OR (Right-Sided Heart Failure)) OR (Myocardial Failure)) OR (Congestive Heart Failure)) OR (Heart Failure, Congestive)) OR (Heart Failure, Left-Sided)) OR (Heart Failure, Left Sided)) OR (Left-Sided Heart Failure)) OR (Left-Sided Heart Failure)) AND ((Shengui capsule) OR (Shengui)) OR (Shen Gui capsule)). In addition, the article reference lists were searched for relevant literature to prevent omissions. The search process is detailed in the Supplementary Material S6, S7.

### 2.3 Data extraction

**FIGURE 1:**
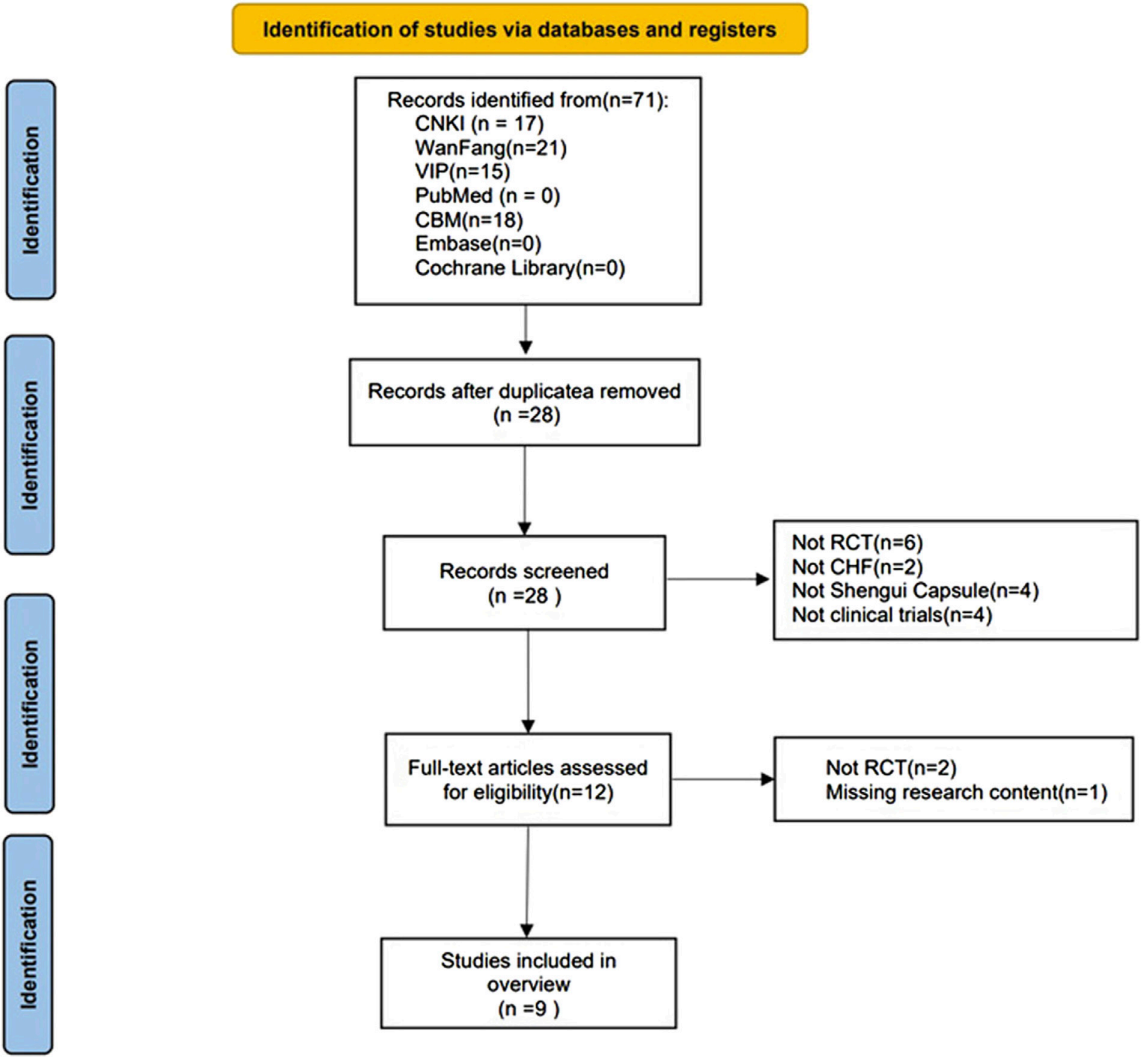
Flow chart of the study selection process.

The literature search and screening were performed independently by two researchers. First, all the literature was searched according to the search strategy and duplicates were eliminated. The titles and abstracts were screened to identify relevant articles. Finally, the full text was read to determine if each article met the inclusion criteria. The researchers discussed any discrepancies, and disagreements were discussed with a third researcher before a final decision was made to include or exclude the article. The screening process is shown in Figure 1.

The data, which were extracted independently by two researchers, included: 1) basic information like article title, first author, nationality, and publication year; 2) logistics like study type, population, interventions, controls, treatment duration, outcomes, and adverse events; and 3) study sample baseline data, including: age, sex, disease duration, comorbidity, and NYHA classification (Table 1). The extracted data were collated and checked by two researchers using Excel 2019; inconsistencies were discussed between these two, or decided by a third researcher. Corresponding authors of articles with incomplete information were contacted; if there was no response, that article was excluded from the study.

**TABLE 1:** Main characteristics of included studies.

	Author (Year)	Country trial design	Study population	Intervention/Comparison	Treatment time	Sample Size (T/C),Dropout (T/C)	Age,Year (T/C)	Gender,No. Of Male (T/C)	Course, Year (T/C)	Comorbidity	NYHA	Outcome assessment
1	Chen Qun2015	China 2-arm	Elderly patients with severe CHF	Benazepril hydrochloride tablets po 2# tid + SGCP po 3# tid**/**Benazepril hydrochloride tablets po 2# tid	4 weeks	98 (49/49),0	70 ± 1.5/71 ± 1.5	27/30	NR	NR	NR	effective rate
2	Cui Ying2018	China 2-arm	Patients with CHF for more than 3 months	Conventional treatment + SGCP po 4# tid**/**Conventional treatment	4 weeks	80 (40/40),0	56.02 ± 5.42/55.68 ± 5.32	23/21	NR	NR	NR	effective rate, hs-CRP, TNF-α, LVESD, LVEDD BNP, LVEF
3	Geng Xiushuang2008	China 2-arm	Severe CHF (NYHA Ⅲ- Ⅳ)	Conventional treatment + SGCP po 3# tid**/**Conventional treatment	4 weeks	68 (34/34),0	70.2 ± 8.1/69.1 ± 8.2	19/18	NR	Experimental group: 16 cases of coronary heart disease, 10 cases of cardiomyopathy, 6 cases of rheumatic heart disease, 2 cases of pulmonary heart disease Control group: 15 cases of coronary heart disease, 9 cases of cardiomyopathy, 7 cases of rheumatic heart disease, 3 cases of pulmonary heart disease	Experimental group: Grade III 20 cases, grade IV 14 cases Control group: 22 cases of grade III and 12 cases of grade IV	effective rate
4	He Xiaoyan2016	China 2-arm	NYHA Ⅱ-Ⅳ	Conventional treatment + SGCP po 4# tid**/**Conventional treatment	4 weeks	180 (90/90),0	62.4 ± 10.5/61.5 ± 9.1	55/51	5.1 ± 2.3/4.8 ± 2.4	NR	Experimental group: 13 cases of grade Ⅱ, 63 cases of grade Ⅲ, 14 cases of grade Ⅳ Control group: 12 cases of grade Ⅱ, 61 cases of grade Ⅲ, 17 cases of grade Ⅳ	effective rate LVEDD LVEF hs-CRP TNF-α BNP
5	Li Songlin2023	China 2-arm	Patients with CHF	Bisoprolol fumarate tablets po 1# qd + SGCP po 4# tid**/**Bisoprolol fumarate tablets po 1# qd	4 weeks	77 (39/38),0	53.90 ± 6.32/53.16 ± 6.74	21/23	1.03 ± 0.33/1.01 ± 0.31	Experimental group: 20 cases of hypertension, 13 cases of coronary heart disease, 6 cases of diabetes Control group: 23 cases of hypertension, 11 cases of coronary heart disease, 4 cases of diabetes	Experimental group: 24 cases of grade Ⅱ and 15 cases of grade Ⅲ Control group: 26 cases of grade Ⅱ and 12 cases of grade Ⅲ	effective rate LVEF BNP
6	Lu Wentao2013	China 2-arm	Patients with CHF; heart-kidney Yang deficiency pattern; NYHA Ⅱ-Ⅲ	Conventional treatment + SGCP po 4# tid**/**Conventional treatment	4 weeks	68 (35/33),0	65 ± 10/66 ± 9	15/14	6.3/6.6	55 cases of coronary heart disease, 36 cases of hypertension, 26 cases of diabetes (no specific group description)	Experimental group: 11 cases of grade Ⅱ, 19 cases of grade Ⅲ, 5 cases of grade IV; Control group: 14 cases of grade Ⅱ, 16 cases of grade Ⅲ, 3 cases of grade IV	effective rate BNP
7	Sang Fengmei2010	China 2-arm	Patients with CHF; NYHA Ⅱ and above; LVEF< 50%	Conventional treatment + SGCP po 3# tid**/**Conventional treatment	4 weeks	40 (21/19),0	Mean 72/74	14/12	0.6-20/0.5-18	Experimental group: Coronary heart disease in 10 cases, pulmonary heart disease in 3 cases, hypertensive heart disease in 8 cases; Control group: 9 cases of coronary heart disease, 2 cases of pulmonary heart disease, 7 cases of hypertensive heart disease, 1 case of dilated cardiomyopathy	NR	effective rate LVEDD LVESD LVEF
8	Yu Chunjuan2013	China 2-arm	Patients with CHF; NYHA Ⅱ-Ⅲ; Yang qi deficiency pattern or blood stasis induced water retention pattern	Conventional treatment + SGCP po 3# tid**/**Conventional treatment	4 weeks	220 (122/98), 0	60.4 ± 7.0/61.4 ± 8.0	75/52	2.34 ± 2.1/2.44 ± 1.7	NR	Experimental group: 67 cases of grade Ⅱ and 55 cases of grade Ⅲ Control group: 55 cases of grade Ⅱ and 43 cases of grade Ⅲ	effective rate hs-CRP
9	Zhuang Rui2021	China 2-arm	Patients with CHF; NYHA Ⅱ-Ⅳa; Yang deficiency and blood stasis pattern	Conventional treatment + SGCP po 4# tid**/**Conventional treatment	8 weeks	57 (30/27),0	79.37 ± 7.62/77.26 ± 10.31	15/15	NR	NR	Experimental group: 14 cases of grade Ⅱ, 12 cases of grade Ⅲ, 4 cases of grade IVa Control group: 13 cases of grade Ⅱ, 9 cases of grade Ⅲ, 5 cases of grade IVa	effective rate LVEF

BNP, brain natriuretic peptide; hs-CRP, hypersensitive C-reactive protein; LVEF, left ventricular ejection fraction; LVEDD, left ventricular end-diastolic dimension; LVESD, left ventricular end-systolic diameter; SGCP, Shen Gui capsule; T/C, data of Trail group/data of Control group; TNP; tumor necrosis factor; NR, not report.

### 2.4 Risk of bias

The risk of bias assessment for study inclusion was conducted independently by two researchers. The criteria were based on the quality assessment criteria recommended in the Cochrane Handbook 2019 (Cumpston et al., 2019) for the Evaluation of Intervention Systems based on seven areas: randomized sequence generation, allocation concealment, blinding of subjects and researchers, blinding of outcome assessors, incomplete outcome data, selective reporting of study results, and other issues. Any disputes were discussed and resolved by a third researcher if agreement could not be reached.

### 2.5 Evidence confidence

Grading of Recommendations Assessment, Development, and Evaluation (GRADE) (Schünemann et al., 2020) was used to assess the quality of evidence of the included studies. The quality rating of the outcome indicators was evaluated in five areas: limitations, inconsistency, indirectness, imprecision, and publication bias. After completion, this process was independently checked by two researchers, and disputes were resolved by a third researcher.

### 2.6 Data analysis

The meta-analysis was performed using Review Manager 5.4 and Stata 12. Relative risk (RR) and mean difference (MD) were used as effect indicators for dichotomous and continuous variables, respectively, with 95% confidence intervals (CI). *p* < 0.05 was considered statistically significant. Heterogeneity was assessed using the χ^2^ and the I^2^ tests. The random-effects model found I2>50, indicating greater heterogeneity among the studies. When I2≤50% indicated less heterogeneity among the studies, a fixed-effects model was applied. If the heterogeneity was large and could not be explained in terms of clinical or methodological heterogeneity, descriptive analysis (i.e., rather than a meta-analysis) was performed. Based on the primary outcome indicators and high heterogeneity, subgroup results were analyzed using the following grouping criteria: 1) mean age of sample (<60 or ≥60 years) and 2) the SGCP dose (9 capsules/day or 12 capsules/day).

### 2.7 Sensitivity analysis

Sensitivity analysis was performed using Stata 12 to assess meta-analysis results stability. When results heterogeneity was high, Review Manager 5.4 was used to compare the results of the new effects and the magnitude of heterogeneity to determine the source of heterogeneity, by deleting the included studies one-by-one and rerunning the meta-analysis.

### 2.8 Publication bias

Publication bias was not assessed because the number of included RCTs <10, rendering meaningless both funnel plots and Egger’s test to assess publication bias.

## 3 Results

### 3.1 Search results

A total of 71 relevant articles were retrieved, including: 17 articles in CNKI; 21 articles in Wan Fang; 15 articles in VIP; and 18 articles in CBM. After removing 43 duplicates and screening the titles, abstracts, and full texts, eight non-RCTs, 2 non-CHD studies, 4 non-SGCP studies, 4 non-clinical trials, and 1 incomplete study were excluded. Thus, a final 9 RCTs (Xiushuang et al., 2008; Fengmei, 2010; Chunjuaan et al., 2013; Wentao et al., 2013; Qun, 2015; Xiaoyan and Wenping, 2016; Ying, 2018; Rui et al., 2021; Songlin et al., 2022) were included herein (Figure 1).

### 3.2 Study characteristics

The 9 included RCTs represented a cumulative 888 patients with CHF(Xiushuang et al., 2008; Fengmei, 2010; Chunjuaan et al., 2013; Wentao et al., 2013; Qun, 2015; Xiaoyan and Wenping, 2016; Ying, 2018; Rui et al., 2021; Songlin et al., 2022). All trials were two-arm, with test and control groups, including 460 participants in the experimental group and 428 in the control group. Sample sizes ranged from 40 to 220, with 21–122 in the experimental group and 19–98 in the control group. Regarding interventions, all control groups were treated with conventional HF therapy such as beta-blockers and ACEI/ARBs; one study (Qun, 2015) used diphenhydramine hydrochloride tablets (two tablets, three times daily), and one (Songlin et al., 2022) used bisoprolol fumarate tablets (one tablet, once daily), while the remaining seven studies (Xiushuang et al., 2008; Fengmei, 2010; Chunjuaan et al., 2013; Wentao et al., 2013; Xiaoyan and Wenping, 2016; Ying, 2018; Rui et al., 2021) did not specify medication or dosage details.

The experimental group was administered SGCP in conjunction with conventional HF therapy. Four studies (Xiushuang et al., 2008; Fengmei, 2010; Chunjuaan et al., 2013; Qun, 2015) administered three capsules, twice daily and five studies (Wentao et al., 2013; Xiaoyan and Wenping, 2016; Ying, 2018; Rui et al., 2021; Songlin et al., 2022) administered four capsules, thrice daily. One study (Rui et al., 2021) had a treatment course of 8 weeks, and the other eight (Xiushuang et al., 2008; Fengmei, 2010; Chunjuaan et al., 2013; Wentao et al., 2013; Qun, 2015; Xiaoyan and Wenping, 2016; Ying, 2018; Songlin et al., 2022) had 4-week treatment courses. All studies were conducted and published in China; basic study characteristics are shown in Table 1.

### 3.3 Risk of bias assessment

**FIGURE 2:**
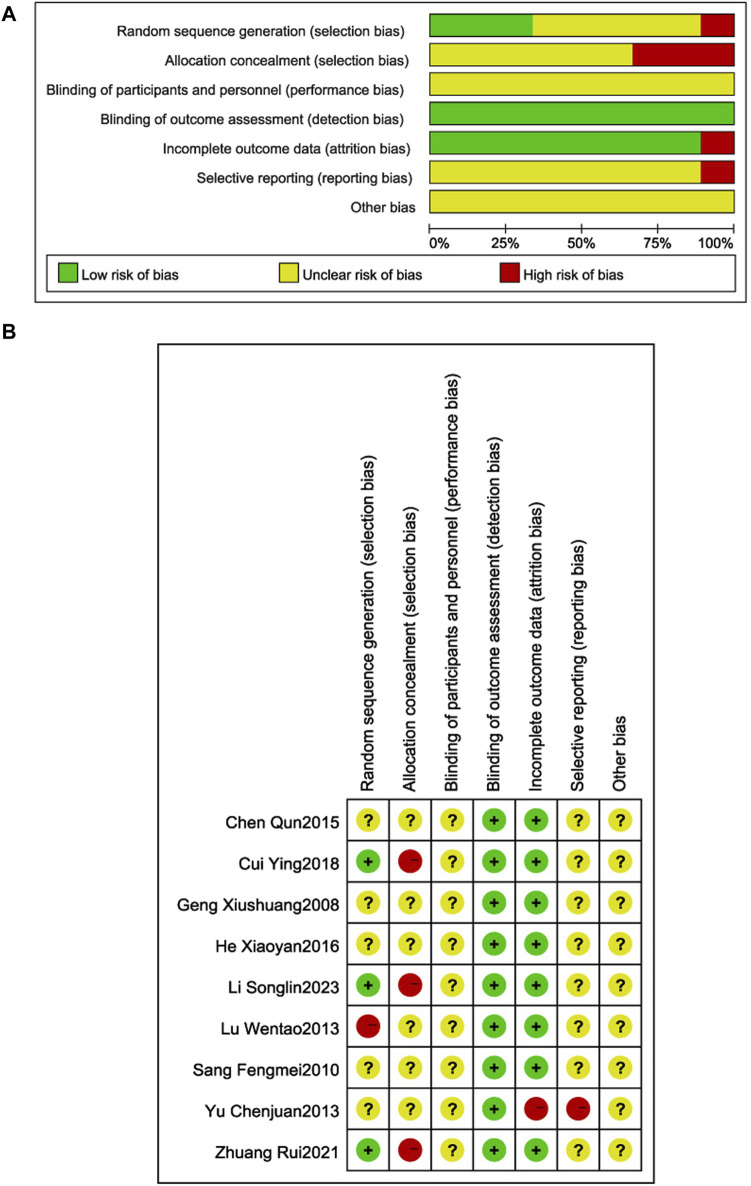
Risk of bias graph and bias summary **(A)**. Risk of bias graph **(B)**. Risk of bias summary.

The quality of the literature was assessed using the Cochrane Risk of Bias Assessment Tool (Figure 2). Three RCTs (Ying, 2018; Rui et al., 2021; Songlin et al., 2022) described the use of random number table method to generate random sequences, and were judged to be low risk; one RCT (Wentao et al., 2013) used a non-random sequence generation method, and was judged to be high risk; the remaining five RCTs (Xiushuang et al., 2008; Fengmei, 2010; Chunjuaan et al., 2013; Qun, 2015; Xiaoyan and Wenping, 2016) did not specify sequence generation details, so the risk of bias judgment was uncertain. Three RCTs (Ying, 2018; Rui et al., 2021; Songlin et al., 2022) were at high risk of selective bias due to the use of open random allocation tables (random number tables) which may have allowed researchers to anticipate allocation; the remaining RCTs did not describe a concealment method, so the risk of bias was uncertain. Patient and researcher blinding was not reported in any of the RCTs, so the risk of implementation bias was uncertain. Blinding of outcome assessors was not reported in any of the RCTs; however, measurement and assessment of outcomes were unaffected, so measurement bias was judged to be low risk. One RCT (Chunjuaan et al., 2013) was judged to be high risk because of incomplete outcome data; the remaining studies were judged to be uncertain because of incomplete information which made it difficult to determine whether there is a risk of selective reporting of results. All RCTs reported insufficient information to judge whether there was a significant risk of bias, so were judged to be uncertain.

### 3.4 Outcomes

#### 3.4.1 Primary outcomes

##### 3.4.1.1 LVEF

**FIGURE 3:**
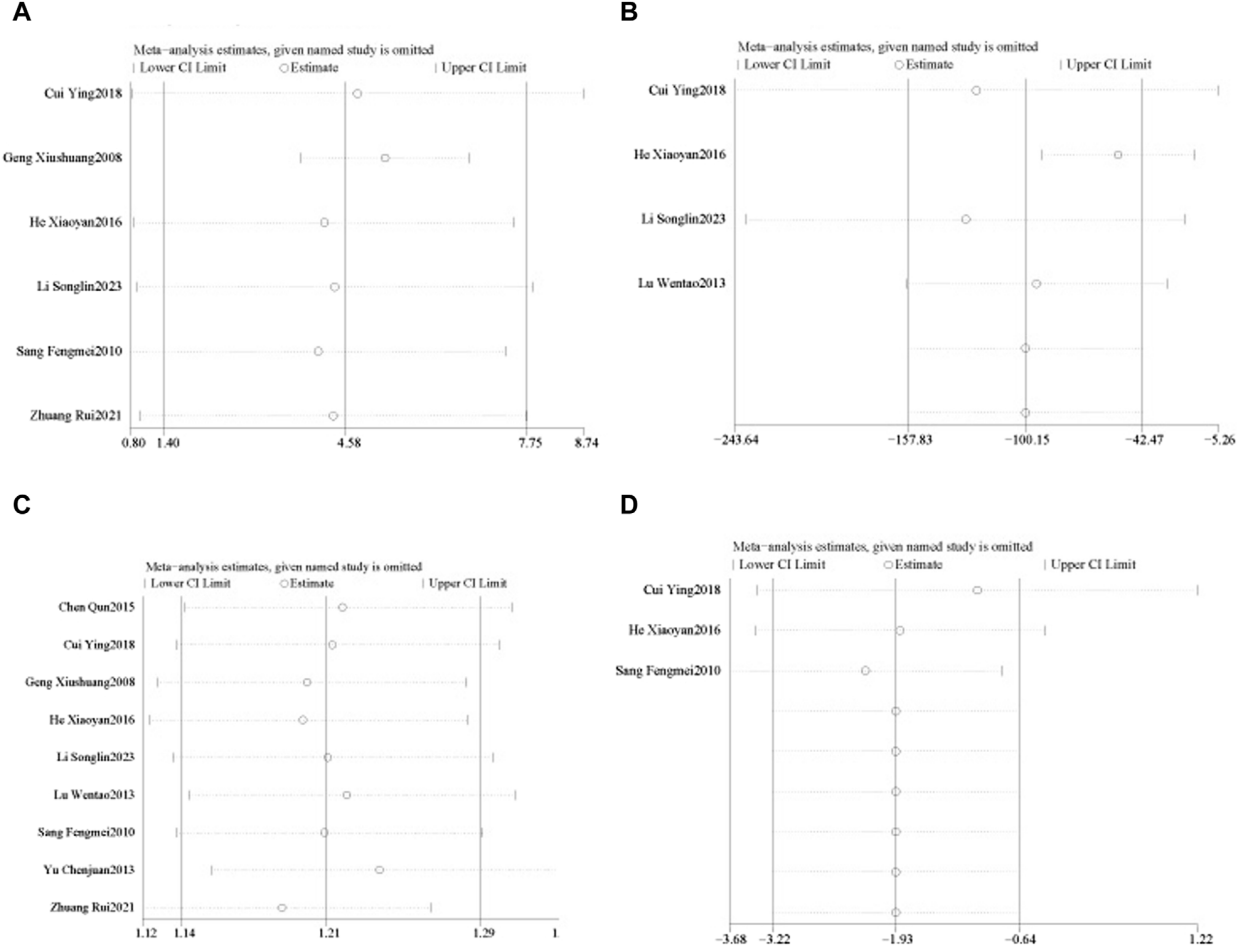
Sensitivity analyses of LVEF, BNP, effective rate, and LVEDD. **(A)**. LVEF; **(B)**. BNP; **(C)**. effective rate; **(D)**. LVEDD.

**FIGURE 4:**
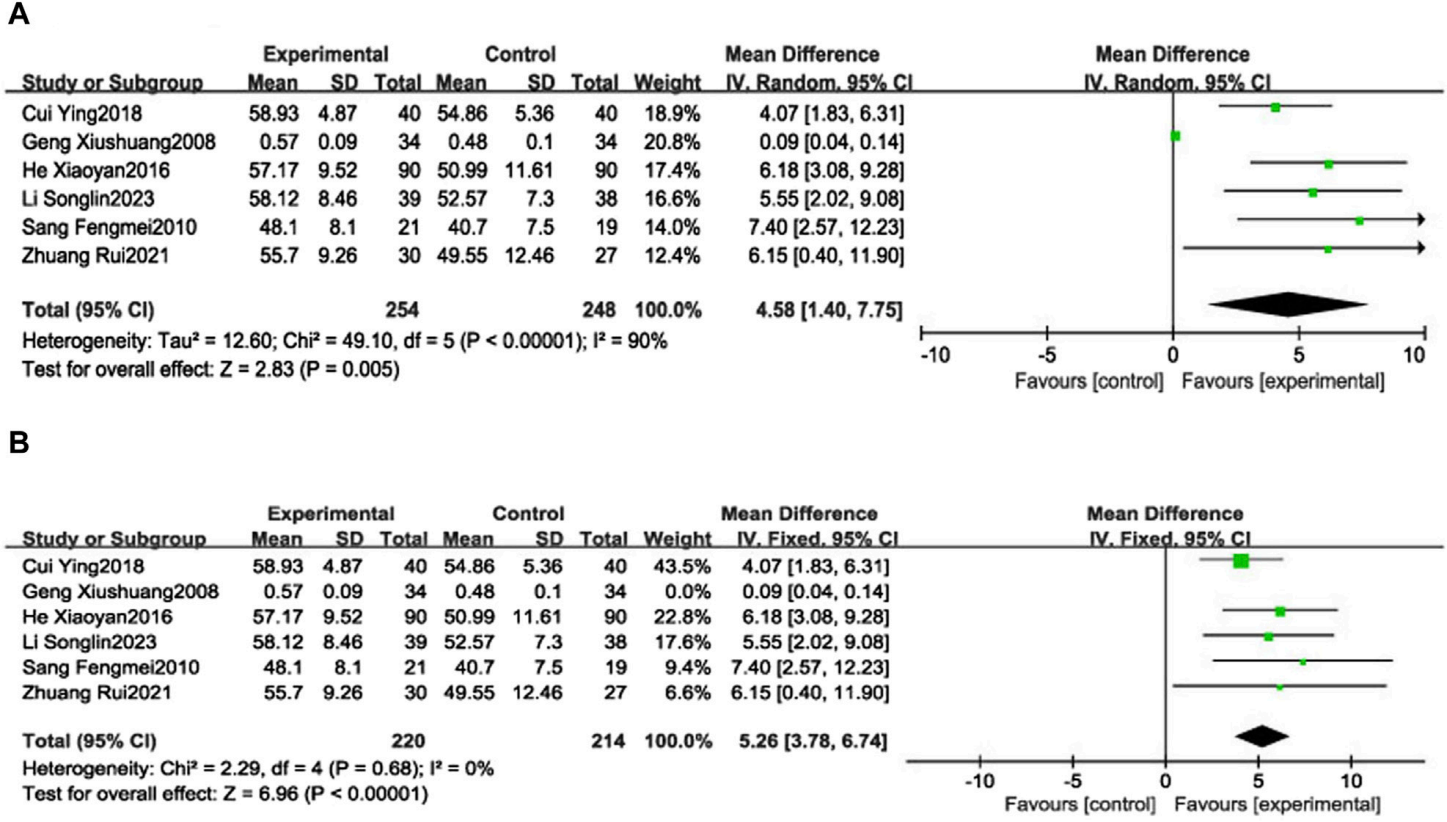
Forest plot of LVEF **(A)**. Forest plot of LVEF **(B)**. Forest plot of LVEF after removing the “Geng Xiushuang 2008” study.

Six RCTs (Xiushuang et al., 2008; Fengmei, 2010; Xiaoyan and Wenping, 2016; Ying, 2018; Rui et al., 2021; Songlin et al., 2022) reported LVEF in 502 patients with CHF, including 248 in the control group receiving conventional therapy and 254 in the experimental group receiving SGCP combined with conventional therapy. Ultimately, there was significant heterogeneity (χ^2^ = 49.10, I^2^ = 90%) among the six RCTs; therefore, a meta-analysis was performed using a random-effects model, revealing that the addition of SGCP to conventional therapy was more conducive to improved LVEF compared with conventional therapy alone [MD = 4.58, 95%CI (1.40, 7.75), *p* = 0.005]. Sensitivity analysis using Stata 12 also suggested a stable outcome, as shown in Figure 3. To explore the source of heterogeneity, the studies were excluded one-by-one in the meta-analysis, with heterogeneity significantly reduced (χ^2^ = 2.29, I^2^ = 0%) after excluding the study by Xiushuang Geng et al. (Xiushuang et al., 2008). That study’s inclusion of patients with NYHA classification III and IV (Table 1) may have been the main factor contributing to the significant heterogeneity. The meta-analysis using a fixed-effects model after excluding this RCT (Xiushuang et al., 2008) suggested that the addition of SGCP to conventional therapy was more beneficial to improving LVEF compared with conventional therapy alone [MD = 5.26, 95%CI (3.78, 6.74), *p* < 0.00001; Figure 4].

Subgroup analyses were performed based on the SGCP dose (9 capsules/day or 12 capsules/day). Two RCTs (Xiushuang et al., 2008; Fengmei, 2010) were included in the low-dose group (9 capsules/day), which reported the LVEF of 108 patients with CHF, including 53 patients in the control group and 55 patients in the experimental group, with significant heterogeneity in the results (χ^2^ = 8.78, I^2^ = 89%). There was not a statistically significant between-groups difference in LVEF [MD = 3.33, 95%CI (−3.79, 10.45), *p* = 0.36]. Four RCTs (Xiaoyan and Wenping, 2016; Ying, 2018; Rui et al., 2021; Songlin et al., 2022) were included in the high-dose group (12 capsules/day), reporting LVEF in 394 patients with CHF, including 195 in the control group and 199 in the experimental group, with no heterogeneity in the results (χ^2^ = 1.46, I^2^ = 0%). This analysis suggested that the addition of SGCP to conventional therapy was more favorable to improving LVEF compared with conventional therapy alone [MD = 5.04, 95%CI (3.49, 6.60), *p* < 0.00001]. Results of age subgroup analyses (<60 or ≥60 years) suggest that this factor is not a source of heterogeneity (Supplementary Material S8).

##### 3.4.1.2 Effective rate

**FIGURE 5:**
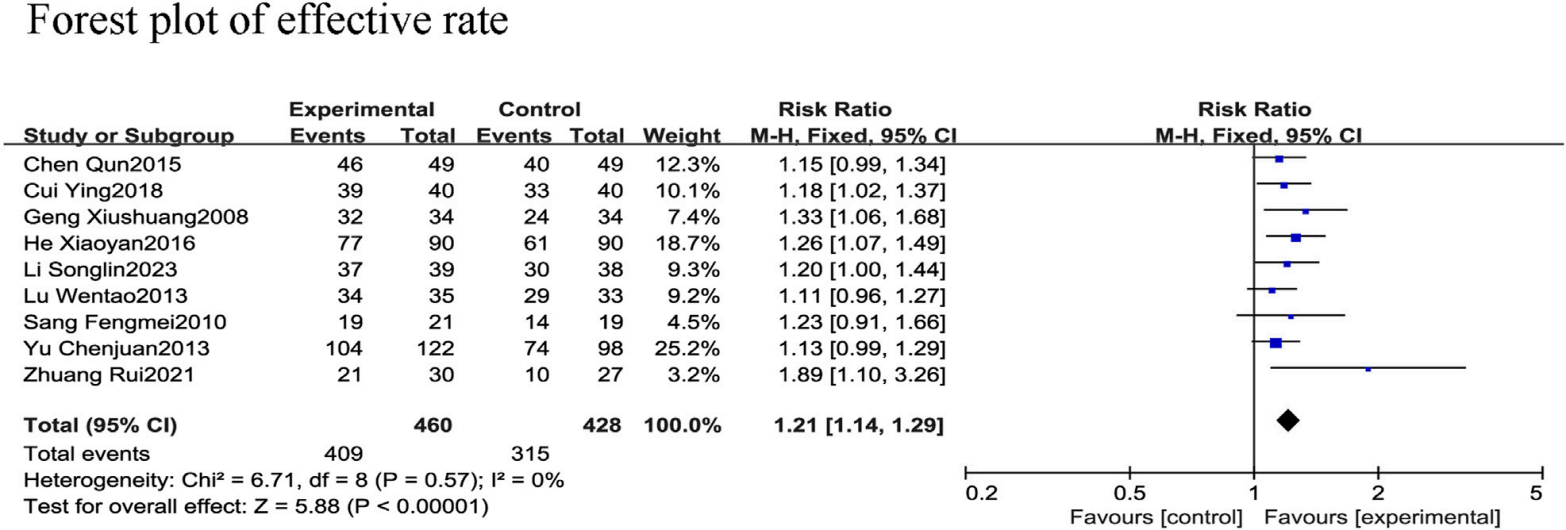
Forest plot of effective rate.

All nine RCTs (Xiushuang et al., 2008; Fengmei, 2010; Chunjuaan et al., 2013; Wentao et al., 2013; Qun, 2015; Xiaoyan and Wenping, 2016; Ying, 2018; Rui et al., 2021; Songlin et al., 2022) reported the effective rate in 888 patients with CHF, including 428 in the control group receiving conventional therapy and 460 in the experimental group receiving SGCP in combination with conventional therapy. There was no heterogeneity among the nine studies (χ^2^ = 6.71, I^2^ = 0%). Therefore, a meta-analysis was performed using a fixed-effect model, revealing that the addition of SGCP to conventional therapy helped to increase the effective rate compared with conventional therapy alone [RR = 1.21, 95%CI (1.14, 1.29), *p* < 0.001; Figure 5]. Sensitivity analysis indicated a stable outcome (Figure 3) and subgroup analyses based on age (<60 or ≥60 years) and SGCP dose (9 or 12 capsules/day) suggested no significant differences based on either factor (Supplementary Material S8).

#### 3.4.2 Secondary outcomes

##### 3.4.2.1 BNP

**FIGURE 6:**
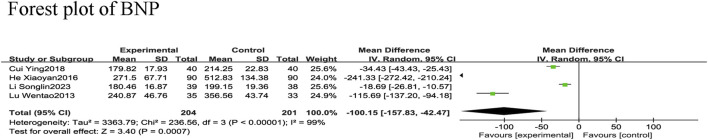
Forest plot of BNP.

Four RCTs (Wentao et al., 2013; Xiaoyan and Wenping, 2016; Ying, 2018; Songlin et al., 2022) reported BNP in 405 patients with CHF, including 201 in the control group and 204 in the experimental group, with significant heterogeneity among studies (χ^2^ = 236.56, I^2^ = 99%). Therefore, a meta-analysis was performed using a random-effects model, revealing that the addition of SGCP to conventional therapy was more conducive to reducing BNP in patients with CHF compared with conventional therapy alone [MD = −100.15, 95%CI (−157.83, −42.47), *p* = 0.0007; Figure 6]. Sensitivity analysis indicated that the overall outcome was stable (Figure 3).

Subgroups were analyzed according to the mean age (<60 or ≥60 years). The age <60 group was included in two RCTs (Ying, 2018; Songlin et al., 2022), reporting 157 patients, with 78 in the control group and 79 in the experimental group. The age ≥60 group was included in two RCTs (Wentao et al., 2013; Xiaoyan and Wenping, 2016), reporting 248 patients, with 123 in the control group and 125 in the experimental group. There was significant heterogeneity in both groups (χ^2^ = 6.48, I^2^ = 85%; χ^2^ = 42.43, I^2^ = 98%, respectively) and the random-effects model revealed that the addition of SGCP to conventional therapy was more beneficial in reducing BNP in patients with CHF compared with conventional therapy alone [MD = −26.44, 95%CI (−41.86, −11.01), *p* = 0.0008; MD = −177.99, 95%CI (−301.11, −54.87), *p* = 0.005, respectively; Supplementary Material S8]. Dose subgroup analyses were not performed because the SGCP dose was the same in these four RCTs (i.e., 12 capsules daily).

##### 3.4.2.2 LVEDD

**FIGURE 7:**
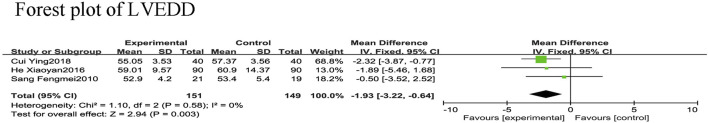
Forest plot of LVEDD.

Three RCTs (Fengmei, 2010; Xiaoyan and Wenping, 2016; Ying, 2018) reported LVEDD in 300 patients with CHF, 149 in the control group and 151 in the experimental group, with no heterogeneity among these studies (χ^2^ = 1.10, I^2^ = 0%). Therefore, a meta-analysis was performed using a fixed-effects model, showing that the addition of SGCP to conventional therapy was more favorable to a reduced LVEDD compared with conventional therapy [MD = −1.93, 95%CI (−3.22, −0.64), *p* = 0.003; Figure 7].

##### 3.4.2.3 hs-CRP

**FIGURE 8:**
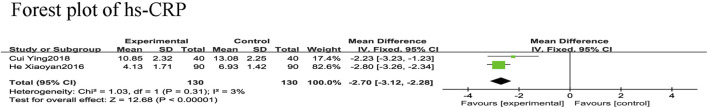
Forest plot of hs-CRP.

Three RCTs (Chunjuaan et al., 2013; Xiaoyan and Wenping, 2016; Ying, 2018) reported hs-CRP in 480 patients with CHF; however, the hs-CRP values in one study (Chunjuaan et al., 2013) were suspected to be incorrect, so those data were excluded. Hs-CRP data of 260 patients with CHF were included, with 130 in the control group and 130 in the experimental group, with slight heterogeneity between the two studies (χ^2^ = 1.03, I^2^ = 3%). Thus, a meta-analysis using a fixed-effect model was performed, revealing that the addition of SGCP to conventional therapy helped to reduce hs-CRP compared with conventional therapy alone [MD = −2.70, 95%CI (−3.12, −2.28), *p* < 0.001; Figure 8].

##### 3.4.2.4 TNF-α

**FIGURE 9:**
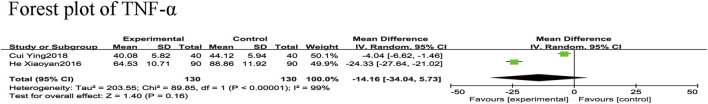
Forest plot of TNF-α

Two RCTs (Xiaoyan and Wenping, 2016; Ying, 2018) reported TNF-α in 260 patients with CHF, including 130 in each of the control and experimental groups, with significant heterogeneity between the studies (χ^2^ = 89.85, I^2^ = 99%). A meta-analysis was performed using a random-effects model, showing that there was not a statistically significant difference in TNF-α levels between SGCP combined with conventional therapy compared with conventional therapy alone [MD = −14.16, 95%CI (−34.04, 5.73), *p* = 0.16; Figure 9). However, inclusion of too few RCTs may have resulted in high heterogeneity.

##### 3.4.2.5 LVESD

**FIGURE 10:**
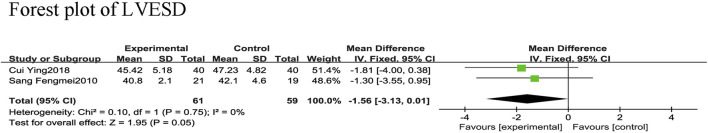
Forest plot of LVESD.

Two RCTs (Fengmei, 2010; Ying, 2018) reported LVESD in 120 patients with CHF, with 59 in the control group and 61 in the experimental group, and no heterogeneity between the two studies (χ^2^ = 0.10, I^2^ = 0%). The fixed-effect model was used for the meta-analysis revealing that SGCP combined with conventional therapy did not significantly reduce LVESD in patients with CHF compared with conventional therapy alone [MD = −1.56, 95%CI (−3.13, 0.01), *p* = 0.05; Figure 10].

### 3.5 Quality of the evidence

None of the included studies described allocation concealment and blinding information in detail, so the evidence was downgraded by one grade in the limitations. The meta-analysis revealed significant heterogeneity in LVEF, TNF-α, and BNP; thus, they were downgraded by two grades in inconsistency. All outcomes could be used as direct evidence, so the indirectness was not downgraded. The LVESD and TNF-α indicators were downgraded by one grade in imprecision because the sample size was <300. LVEF and BNP were downgraded for potential publication bias due to funnel plot asymmetry. The effective rate, hs-CRP, and LVEDD indexes were rated as “intermediate” quality evidence, LVESD was rated as “low”, and LVEF, TNF-α, and BNP were rated as “very low” (Table 2).

**TABLE 2:** Quality of evidence.

Outcomes	Sample Size (T/C)	Limitations	Inconsistency	Indirectness	Imprecision	Publication bias	Relative effect (95% CI)	I2(%)	Quality
Effective rate	9 (888)	−1^a^	0	0	0	0	RR = 1.21 (1.14, 1.29)	0	Moderate
LVEF	6 (502)	−1^a^	−2^b^	0	0	−1^d^	MD = 4.58 (1.40, 7.75)	90	Very low
hs-CRP	3 (480)	−1^a^	0	0	0	0	MD = −2.70 (−3.12, −2.28)	3	Moderate
TNF-α	2 (260)	−1^a^	−2^b^	0	−1^c^	0	MD = −14.16 (−34.04, 5.73)	99	Very low
LVESD	2 (120)	−1^a^	0	0	−1^c^	0	MD = −1.56 (−3.12, 0.01)	0	low
LVEDD	3 (300)	−1^a^	0	0	0	0	MD = −1.93 (−3.22, −0.64)	0	Moderate
BNP	4 (405)	−1^a^	−2^b^	0	0	−1^d^	MD = −100.15 (−157.83, −42.47)	99	Very low

BNP, brain natriuretic peptide; LVEDD, left ventricular end-diastolic dimensions; LVEF, left ventricular ejection fraction; LVESD, left ventricular end-systolic dimensions.

^a^ The included study had an unclear risk of selection, performance, detection, and reporting biases.

^b^ 50 ≤ I2 < 75%.

^c^ Sample size <300.

^d^ There may be publication bias.

### 3.6 Adverse events

Five RCTs (Fengmei, 2010; Wentao et al., 2013; Qun, 2015; Xiaoyan and Wenping, 2016; Ying, 2018) did not report adverse events and two (Chunjuaan et al., 2013; Rui et al., 2021) reported no significant adverse events. In one RCT (Xiushuang et al., 2008), five patients in each of the experimental and control groups experienced generalized fever. In the other study (Songlin et al., 2022), there were two reported episodes of headaches, one of dizziness, and one of dry mouth and thirst in the control group (incidence = 10.53%), and in the experimental group there was one episode of headache, two of dizziness, one of fatigue, and two of dry mouth and thirst (incidence = 15.38%). There was not a significant between-groups difference in the incidence of adverse events (*p* > 0.05).

## 4 Discussion

This meta-analysis of data from nine RCTs, representing a cumulative 888 patients with CHF, found that conventional therapy combined with SGCP was more conducive to improving LVEF, increasing treatment efficiency, and decreasing BNP, LVEDD, and hs-CRP compared with conventional therapy alone; however, there was not a significant difference in reduced TNF-α or LVESD.

The quality of the included studies and evidence varied, with the quality of outcomes rated as intermediate-to-very-low, and no high-quality evidence studies. The main reasons for downgrading were inconsistency, limitations, and imprecision. Heterogeneity of LVEF, TNF-α, and BNP were all significant. Heterogeneity reflects widely varying estimates of treatment effects across individual studies, indicating real differences in potential treatment effects (Yang et al., 2023); thus, higher heterogeneity in outcomes across studies is downgraded due to inconsistency across results. In terms of limitations, no study described specific information on allocation concealment and blinding, thereby reducing the quality of evidence assessment based on the study design, which may lead to biased estimates of treatment effects (Wang et al., 2024). In terms of imprecision, when a study sample size is small, CIs for outcomes tend to be wider, contributing to uncertainty of the results (Guyatt et al., 2023). Consequently, the quality of the evidence for LVESD and TNF-α was downgraded, and that for effective rate, hs-CRP, and LVEDD, which were intermediate-quality evidence, had high confidence in the outcome estimation. However, further high-quality studies are required to confirm the effect of conventional treatment combined with SGCP on LVEF, TNF-α, BNP, and LVESD.

Regarding the quality of the literature, in terms of randomization, unbiased interventional studies need to ensure that similar participants receive respective interventions according to the principle of random assignment. Herein, five RCTs (Xiushuang et al., 2008; Fengmei, 2010; Chunjuaan et al., 2013; Qun, 2015; Xiaoyan and Wenping, 2016) did not describe sequence-generated information to determine the risk of bias, which raised the possibility of bias in the allocation of interventions. Intervention assignment in one RCT (Wentao et al., 2013) during sequence generation resulted in a high risk of selective bias. All these factors affect the overall literature quality. In terms of allocation sequence concealment, three RCTs (Ying, 2018; Rui et al., 2021; Songlin et al., 2022) used randomized number tables which may lead to selective recruitment based on prognostic factors. This open random assignment method did not accomplish adequate concealment of participant sequences and was prone to a high risk of selective bias. In addition, none of the other RCTs described the exact method of assigning sequence hiding. A study (Wood et al., 2008) of 146 meta-analyses found that in trials with subjective endpoints where allocation concealment was inadequate or unclear, there might be a component of amplification of the effect of their interventions on outcomes, thereby increasing the risk of bias. In addition, incomplete information made it impossible to determine whether there is a risk of selective reporting of results and the possibility of causing other biases, which would lead to a potential risk of bias in the reported results, which in turn would affect the study results.

Furthermore, none of the RCTs herein reported blinding of patients or outcome assessors. It has been shown that in randomized trials with ambiguous blinding or lack of blinding, the value of their intervention effect on outcomes was exaggerated from the estimated value (Tack, 2023). In general, the more subjective the trial outcome, the greater the bias it creates (Wang et al., 2023). However, the risk of measurement bias was low herein because the outcomes were objective and did not influence the measurement and assessment of the outcome. The data from only one RCT (Chunjuaan et al., 2013) was suspected to be incorrect, which would increase the likelihood of bias in the observed effect estimates. Therefore, these data were excluded to prevent such bias. All RCTs reported all outcomes and there was no risk due to selective reporting. Nonetheless, the studies might also be at risk of bias because of factors such as specific design, baseline imbalance, block randomization of unblinded trials, and varying diagnostic activity; however, no other sources of bias were identified in any of the included RCTs, partly maintaining their quality.

Sensitivity analyses revealed that all the results were robust. The main heterogeneity sources are clinical and methodological (Yalla and Lambrechts, 2023). To assess clinical heterogeneity, we performed subgroup analyses based on mean patient age (<60 or ≥60 years) and SGCP dose (9 capsules/day or 12 capsules/day), finding that neither was a source of heterogeneity. This was explored by excluding single studies on a one-by-one basis to re-run the meta-analysis, showing that patient NYHA classification may have been a primary source of heterogeneity for LVEF. Heterogeneity was reduced by excluding that study. In addition, most included studies did not mention the specific drugs and methods of administration of conventional therapy, and factors such as varying levels of medical care across study centers may have contributed to clinical heterogeneity, which was unavoidable due to the limitations of the objective factors. Methodological heterogeneity might have resulted from including evidence quality ranging from intermediate to very low, and risk of bias in the quality of the literature. Therefore, more studies with higher-quality evidence and higher-quality literature are needed to reduce these heterogeneities.

Characteristics of CHF include dyspnea or limitations in exercise due to impaired ventricular filling or ejection (Metra et al., 2023). Therefore, LVEF measured by echocardiography and serum BNP are important indicators in CHF diagnosis. HF with reduced ejection fraction occurs when the LVEF is ≤ 40%, and is accompanied by progressive left ventricular dilatation and ventricular remodeling (Murphy et al., 2020); this context increases LVEDD and LVESD. BNP is mainly secreted by ventricular myocytes, and promotes excretion, urination, and vasodilatation. When the ventricular load and ventricular wall tension are altered, BNP secretion is promoted to reduce cardiac load and protect cardiac function (Kuwahara, 2021). Therefore, when BNP is elevated above age- and underlying disease-specific thresholds, the potential for HF diagnosis increases accordingly. Moreover, because BNP is an effective predictor of death and acute cardiovascular events at 2–3 months (Yazdani et al., 2023), it can be used to assess cardiac function and long-term prognosis among patients with CHF. As an inflammatory biomarker, hs-CRP is associated with worsening CHF pathogenesis (Magnussen and Blankenberg, 2018). In a prospective cohort study (Burger et al., 2023) of patients with cardiovascular disease, hs-CRP was an independent risk marker for HF development. Therefore, hs-CRP levels may reflect severity and prognosis among patients with CHF. Clinical studies (Hanna and Frangogiannis, 2020) have reported significant TNF-α levels in patients with CHF, and mice with cardiac-specific TNF-α overexpression were more likely to develop dilated cardiomyopathy (Zhang and Dhalla, 2024). This mechanism may be related to TNF-α stimulating the synthesis of other pro-inflammatory cytokines with pro-apoptotic and negative inotropic properties, which leads to ventricular remodeling and HF progression (Galeone et al., 2023). Therefore, TNF-α also reflects disease severity in patients with CHF. These outcomes were chosen to assess treatment effectiveness herein to provide a reference basis for the efficacy of SGCP in combination with conventional HF therapy.

SGCP contains *Panax ginseng* C.A.Mey [ Araliaceae; ginseng radix et rhizoma], *Oreocome striata* (DC.) Pimenov and Kljuykov [ Apiaceae; chuanxiong rhizoma], and *Neolitsea cassia* (L.) Kosterm [Lauraceae; cinnamomi ramulus] with the main active metabolites of ginsenoside, ligustrazine, and cinnamaldehyde. Modern pharmacology (Yating et al., 2018) has demonstrated that ginsenoside Rb1 in *P. ginseng* C.A.Mey. Improves ventricular remodeling in CHF rats by affecting the expression of periostin proteins in myocardial tissues and inhibiting the TGF-β signaling pathway to slow CHF progression. Ligustrazine in *O. striata* (DC.) Pimenov and Kljuykov. Exerts anti-apoptotic effects and protects cardiomyocytes by regulating phosphatidylinositol 3-kinase/protein kinase B (Li et al., 2023), and inhibits the JAK kinase/signal transducer activator of transcription signaling pathway of hypertrophic cardiomyocytes to improve ventricular remodeling (Huajuan et al., 2023). Cinnamaldehyde in *N. cassia* (L.) Kosterm. Inhibits the release of histamine and prostaglandin E, and scavenges excessive oxygen free radicals to protect myocardial cell membranes and prevent myocardial damage (Chang et al., 2023). Substances extracted from *N. cassia* (L.) Kosterm. can reduce the expression of RhoA and ROCK2, and inhibit the phosphorylation of target molecules downstream of ROCK, thereby dilating central and peripheral blood vessels and reducing cardiac load (Liu et al., 2020). Different classes and sources of effective metabolites in SGCP can achieve multi-component, multi-pathway, and multi-target therapeutics through synergistic or complementary effects (Ning, 2019). Pharmacological experiments (Qian et al., 2018) have shown that SGCP can significantly improve the energy metabolism of the myocardium in rats with cardiac insufficiency after acute myocardial infarction, protect the myocardial mitochondria from lipid peroxidation, and reduce the content of plasma ET and Ang Ⅱ, improving the systolic and diastolic functions of the myocardium. SGCP may improve myocardial energy metabolism by reducing methylmalonic acid accumulation (Wang et al., 2022). In summary, SGCP can improve cardiac function through multiple pathways and has certain advantages in CHF treatment.

Four of the nine RCTs included herein reported adverse events in the SGCP and control groups, without a significant between-groups difference in these incidences. Therefore, SGCP can be considered safe for CHF treatment, with few side effects. At the same time, other clinical studies (Guo and Zhong, 2006; Zhou, 2016; Li, 2017) on SGCP have also proved that its liver and kidney functions before and after treatment are at normal levels. However, these studies were not conducted in recent years, and the side effects of many traditional Chinese medicines are still unclear due to the complex metabolites, resulted it difficult to explore. In addition, the short period of time in which the study was conducted may have contributed to the fact that no significant side effects were reported. Therefore, these reports of adverse events also indicate that the safety of SGCP needs further evaluation, in more high-quality studies.

### 4.1 Research implications

Based on these cumulative results, we recommend three areas for future studies of SGCP efficacy and safety in CHF treatment. First, regarding study design, random sequence generation and allocation should be strictly controlled, to reduce selective bias, and all researchers and participants should be blinded. Second, more trials comparing the efficacy of SGCP with other Chinese patent medicines in CHF treatment are needed. Finally, future studies of CHF treatment with SGCP should evaluate adverse reactions. Although most current trials have not reported serious adverse reactions, larger studies are required for verification.

### 4.2 Limitations

This study was not without limitations. First, the number of RCTs included was small, and the quality of evidence for some outcomes was not high; therefore, further high-quality RCTs are required to confirm the results. Second, because SGCP is a TCM compound, there were no international studies, and the included studies were all published in Chinese; thus, it is unknown whether CHF treatment with SGCP is affected by factors such as ethnicity, and multicenter international studies are required.

### 4.3 Conclusion

SGCP combined with conventional therapy can improve cardiac ejection function, increase treatment efficacy, and improve HF in patients with CHF, and has certain safety. However, low quality of current evidence means that further high-quality studies are needed to confirm the effectiveness of this treatment.

## Data Availability

The original contributions presented in the study are included in the article/Supplementary Material, further inquiries can be directed to the corresponding authors.

## Abbreviations

ACEI, angiotensin-converting enzyme inhibitor; ARB, angiotensin AT-1 receptor blocker; BNP, brain natriuretic peptide; CBM, China Biology Medicine; CHIs, Chinese herbal injections; CHF, chronic heart failure; ConPhyMP, consensus statement on the Phytochemical Characterisation of Medicinal Plant extract; CI, confidence interval; CNKI, China National Knowledge Infrastructure; GRADE, grading of recommendations assessment, development, and evaluation; HF, heart failure; hs-CRP, hypersensitive C-reactive protein; LVEDD, left ventricular end-diastolic diameter; LVEF, left ventricular ejection fraction; LVESD, left ventricular end-systolic diameter; MD, mean difference; NYHA, New York Heart Association; PRISMA, preferred reporting items for systematic reviews and meta-analyses; RCT, randomized controlled trial; RR, relative risk; SGCP, Shen Gui capsule; TCM, traditional Chinese medicine; TNF-α, tumor necrosis factor-α; VIP, China Science and Technology Journal Database.
