# Supplementary material for: Efficacy and safety of Shen Gui capsules for chronic heart failure: a systematic review and meta-analysis
Source: Front Pharmacol. 2024 Apr 10;15:1347828. doi: 10.3389/fphar.2024.1347828 (PMC11039789; doi:10.3389/fphar.2024.1347828)
Supplement: Supplementary file 1 [file DataSheet1.pdf]

Efficacy and Safety of Shen Gui capsules for Chronic Heart Failure: A Systematic Review and Meta-analysis

Supplementary Material

Table of contents

Supplementary File S1. Composition of the ShenGui capsule....... 1

Supplementary File S2. Extract and extraction process description of the Shen Gui capsule....... 2

Supplementary File S3. Details about the product information of Shengui capsule....... 3

Supplementary File S4. The identification process of Shen Gui capsule....... 3

Supplementary File S5. PRISMA 2020 checklist....... 4

Supplementary File S6. Search strategies for databases....... 8

Supplementary File S7. Literature screening process....... 23

Supplementary File S8. Results of subgroup analysis 26

Supplementary File S1. Composition of the ShenGui capsule

Chinese drug name	Botanical plant name	Family	Plant part used	Component ingredients to be measured
Hongshen	*Panax ginseng* C.A.Mey.	Araliaceae	Root and rhizome	Ginsenoside
Chuanxiong	*Oreocome striata* (DC.) Pimenov & Kljuykov.	Apiaceae	Rhizome	Ligustrazine
Guizhi	*Neolitsea cassia* (L.) Kosterm.	Lauraceae	Twigs	Cinnamaldehyde

Supplementary File S2.Extract and extraction process description of the Shen Gui capsule.

The extract and extraction process descripyion of the ShenGui capsule are as follows:

1. Take 200g of *Panax ginseng* C.A.Mey., pulverized into fine powder.
2. Crush the remaining *Panax ginseng* C.A.Mey. into coarse powder, with 65% ethanol as solvent for percolation and collect the percolating liquid about 2000ml.
3. *Oreocome striata* (DC.) Pimenov & Kljuykov., *Neolitsea cassia* (L.) Kosterm. distillation with water to extract volatile oil, the distillate filtered to get the distillate and volatile oil.
4. The dregs were decocted with the *Panax ginseng* C.A.Mey. dregs in water for two times, each time for 1 hour, and then filtered. The filtrate was combined and concentrated to a clear paste with a relative density of 1.03~1.08 (60C).
5. Put the clear paste after cooling, add ethanol to make the alcohol content of 65%, more than 24 hours after filtration.
6. Combine the filtrate with *Panax ginseng* C.A.Mey. percolating solution, recover ethanol and concentrate to thick paste, add 150g of *Panax ginseng* C.A.Mey. powder, mix and dry, and then crush into fine powder.
7. Adsorb the volatile oil with the remaining *Panax ginseng* C.A.Mey. powder, mix well, then mix with the powder, add starch and mix well, put into the capsule and make 1000 capsules.

Supplementary File S3.The identification process of Shen Gui capsule.

The identification process of SGCP is as follows:

(1) Take this capsule, put it under the microscope to observe, the resin tract fragments are easy to see, containing brownish-yellow lumpy secretion, calcium oxalate cluster crystals with a diameter of 20~68 pm, and its angles are sharp (*Panax ginseng* C.A.Mey.).

(2)Take 2g of the contents of this capsule, add dichloromethane 40ml, put on a water bath and heat reflux for 30 minutes, filtration filtrate standby. The dregs of the drug evaporate the solvent, add 1ml of water, mix well to make wet, add water saturated n-butanol 20ml, ultrasonic treatment for 30 minutes. They were filtered, and the filtrate was washed with n-butanol saturated ammonia solution twice, 20 ml each time, and combined with the n-butanol solution, evaporated to dryness, and the residue was added with 1 ml of methanol to make it dissolved, as the test solution. Take 1g of Panax ginseng C.A.Mey. control botanical drug, and make control botanical drug solution by the same method. Then take ginsenoside Rgr control, ginsenoside Re control, ginsenoside Rbr control, add methanol to make a mixed solution containing 1mg per 1ml, as control solution. According to the thin-layer chromatography (General rule 0502) test, absorb the above three solutions each 1pl, respectively, point in the same silica gel G thin-layer plate, trichloromethane-ethyl acetate-methanol-water (15:40:22:10) 10℃ placed below the lower layer of the solution as the unfolding agent. They were removed and dried, sprayed with 10% ethanol sulfate solution, heated at 105C until the spots showed clear color, and examined under daylight and UV light (365nm) respectively. In the chromatogram of the test capsule, in the corresponding position with the chromatogram of the control botanical drug and the control chromatogram, the same color spots under sunlight; fluorescent spots of the same color under ultraviolet light.

(3) Take the above (2) under the di-gas methane spare filtrate, at a temperature of 60 ℃ concentrated to 1 ml, as a test solution. Another take Oreocome striata (DC.) Pimenov & Kljuykov. control botanical drug 0.3 g, add dichloromethane 15 ml, ultrasonic treatment for 15 minutes, filtration, filtrate at 60 ℃ concentrated to 1 ml, as the control botanical drug solution. Then take cinnamaldehyde control capsule, add methylene chloride to make a solution containing 1ul per 1ml, as the control solution. According to thin-layer chromatography (General rule 0502), absorb 5μl of the test solution, 2μl of the control solution, 5pl of the control solution, were spotted on the same silica gel G thin-layer plate, n-hexane ethyl acetate (9: 1) as an unfolding agent, unfolding, take out, drying, and placed in the ultraviolet lamp (365 nm) under the examination. In the chromatogram of the test material, in the corresponding position with the chromatogram of the control material, the fluorescent main spot of the same color was shown; sprayed with dinitrophenyl hydrazine ethanol test solution, and examined under the sunlight, in the corresponding position with the chromatogram of the control material, the spot of the same color was shown.

Supplementary File S4.Details about the product information of Shen Gui capsule

Capsule name	Source	Therapeutic claims in TCM	Indications	Adverse drug reactions	Quality control reported? (Y/N)	Chemical analysis is reporter? (Y/N)
Shengui Capsule	Shanghai Yudan Pharmaceutical Co.	Promoting Qi and Yang, activating blood circulation and removing blood stasis.	The symptoms are stabbing pain in the chest, fixed and immovable, worse at night, aggravated by cold, or fear of cold and like warmth, less colorful or coronary heart disease angina pectoris with the above symptoms.	There is no description about the side effects in the Chinese Pharmacopoeia.	Z20000060 issued in 2019 by National Medical Products Administration of China	N

Supplementary File S5. PRISMA 2020 checklist

Section and Topic	Item #	Checklist item	Location and whether item is reported
**TITLE**			
Title	1	Identify the report as a systematic review.	Yes
**ABSTRACT**			
Abstract	2	See the PRISMA 2020 for Abstracts checklist.	Yes
**INTRODUCTION**			
Rationale	3	Describe the rationale for the review in the context of existing knowledge.	Yes
Objectives	4	Provide an explicit statement of the objective(s) or question(s) the review addresses.	Yes
**METHODS**			
Eligibility criteria	5	Specify the inclusion and exclusion criteria for the review and how studies were grouped for the syntheses.	Yes
Information sources	6	Specify all databases, registers, websites, organisations, reference lists and other sources searched or consulted to identify studies. Specify the date when each source was last searched or consulted.	Yes<br>Supplementary file S6 and S7
Search strategy	7	Present the full search strategies for all databases, registers and websites, including any filters and limits used.	Yes<br>Supplementary file S6 and S7
Selection process	8	Specify the methods used to decide whether a study met the inclusion criteria of the review, including how many reviewers screened each record and each report retrieved, whether they worked independently, and if applicable, details of automation tools used in the process.	Yes

Data collection process	9	Specify the methods used to collect data from reports, including how many reviewers collected data from each report, whether they worked independently, any processes for obtaining or confirming data from study investigators, and if applicable, details of automation tools used in the process.	Yes
Data items	10a	List and define all outcomes for which data were sought. Specify whether all results that were compatible with each outcome domain in each study were sought (e.g. for all measures, time points, analyses), and if not, the methods used to decide which results to collect.	Yes
	10b	List and define all other variables for which data were sought (e.g. participant and intervention characteristics, funding sources). Describe any assumptions made about any missing or unclear information.	Yes
Study risk of bias assessment	11	Specify the methods used to assess risk of bias in the included studies, including details of the tool(s) used, how many reviewers assessed each study and whether they worked independently, and if applicable, details of automation tools used in the process.	Yes
Effect measures	12	Specify for each outcome the effect measure(s) (e.g. risk ratio, mean difference) used in the synthesis or presentation of results.	Yes
Synthesis methods	13a	Describe the processes used to decide which studies were eligible for each synthesis (e.g. tabulating the study intervention characteristics and comparing against the planned groups for each synthesis (item #5)).	Supplementary file S7
	13b	Describe any methods required to prepare the data for presentation or synthesis, such as handling of missing summary statistics, or data conversions.	Yes
	13c	Describe any methods used to tabulate or visually display results of individual studies and syntheses.	Yes
	13d	Describe any methods used to synthesize results and provide a rationale for the choice(s). If meta-analysis was performed, describe the model(s), method(s) to identify the presence and extent of statistical heterogeneity, and software package(s) used.	Yes
	13e	Describe any methods used to explore possible causes of heterogeneity among study results (e.g. subgroup analysis, meta-regression).	Yes
	13f	Describe any sensitivity analyses conducted to assess robustness of the synthesized results.	Yes
Reporting bias assessment	14	Describe any methods used to assess risk of bias due to missing results in a synthesis (arising from reporting biases).	Yes
Certainty	15	Describe any methods used to assess certainty (or confidence) in the body of evidence for an outcome.	Yes

assessment			
**RESULTS**			
Study selection	16a	Describe the results of the search and selection process, from the number of records identified in the search to the number of studies included in the review, ideally using a flow diagram.	Yes
	16b	Cite studies that might appear to meet the inclusion criteria, but which were excluded, and explain why they were excluded.	Yes<br>Supplementary file S7
Study characteristics	17	Cite each included study and present its characteristics.	Yes
Risk of bias in studies	18	Present assessments of risk of bias for each included study.	Yes
Results of individual studies	19	For all outcomes, present, for each study: (a) summary statistics for each group (where appropriate) and (b) an effect estimate and its precision (e.g. confidence/credible interval), ideally using structured tables or plots.	Yes
Results of syntheses	20a	For each synthesis, briefly summarise the characteristics and risk of bias among contributing studies.	Yes,TABLE1
	20b	Present results of all statistical syntheses conducted. If meta-analysis was done, present for each the summary estimate and its precision (e.g. confidence/credible interval) and measures of statistical heterogeneity. If comparing groups, describe the direction of the effect.	Yes
	20c	Present results of all investigations of possible causes of heterogeneity among study results.	Yes
	20d	Present results of all sensitivity analyses conducted to assess the robustness of the synthesized results.	Yes
Reporting biases	21	Present assessments of risk of bias due to missing results (arising from reporting biases) for each synthesis assessed.	Not reported
Certainty of evidence	22	Present assessments of certainty (or confidence) in the body of evidence for each outcome assessed.	Yes,TABLE2
**DISCUSSION**			

Discussion	23a	Provide a general interpretation of the results in the context of other evidence.	Yes
	23b	Discuss any limitations of the evidence included in the review.	Yes
	23c	Discuss any limitations of the review processes used.	Yes
	23d	Discuss implications of the results for practice, policy, and future research.	Yes
**OTHER INFORMATION**			
Registration and protocol	24a	Provide registration information for the review, including register name and registration number, or state that the review was not registered.	Yes
	24b	Indicate where the review protocol can be accessed, or state that a protocol was not prepared.	Yes
	24c	Describe and explain any amendments to information provided at registration or in the protocol.	Yes
Support	25	Describe sources of financial or non-financial support for the review, and the role of the funders or sponsors in the review.	Yes
Competing interests	26	Declare any competing interests of review authors.	Yes
Availability of data, code and other materials	27	Report which of the following are publicly available and where they can be found: template data collection forms; data extracted from included studies; data used for all analyses; analytic code; any other materials used in the review.	Yes

From: Page MJ, McKenzie JE, Bossuyt PM, Boutron I, Hoffmann TC, Mulrow CD, et al. The PRISMA 2020 statement: an updated guideline for reporting systematic reviews. BMJ 2021;372:n71. doi: 10.1136/bmj.n71

For more information, visit: http://www.prisma-statement.org/

Supplementary File S6. Search strategies for databases.

1. China National Knowledge Infrastructure (CNKI)

The database search in CNKI was carried out on January 21, 2023, and a total of 17 literatures were found.

search strategy

SU=('心力衰竭'+'心衰'+'心功能不全')*'参桂胶囊'

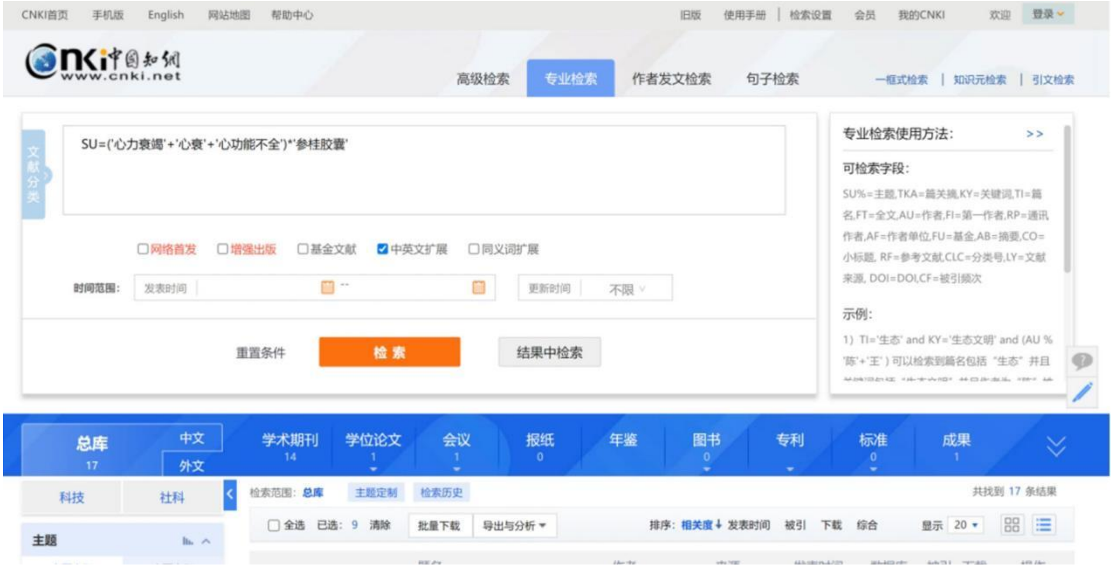

Supplementary Figure 1. Search strategy of CNKI

[1] 李松林,谷朝华,马涛,姚先丽.参桂胶囊联合比索洛尔治疗慢性心力衰竭的临床研究[J/OL].现代药物与临床:1-4.http://kns.cnki.net/kcms/detail/12.1407.R.20221228.2204.006.html

[2]庄锐,吴旸,常佩芬,刘小芸,贺小芳,王尔玉,魏大为,张玲霞,朱海燕.参桂胶囊联合西药治疗冠心病慢性心力衰竭阳虚血瘀证患者临床疗效研究[J].辽宁中医药大学学报,2021,23(06):54-58.DOI:10.13194/j.issn.1673-842x.2021.06.013.

[3]庄锐. 参桂胶囊治疗冠心病慢性心力衰竭阳虚血瘀证患者临床疗效研究[D].北京中医药大学,2019.

[4]崔莹.参桂胶囊治疗慢性心力衰竭的临床研究[J].中西医结合心脑血管病杂志,2018,16(18):2670-2672.

[5]何潇言,严文萍.参桂胶囊治疗慢性心力衰竭的疗效及对BNP的影响研究[J].现代中西医结合杂志,2016,25(14):1512-1514.

[6]俞春娟, 参桂胶囊在慢性充血性心力衰竭中的应用及对神经内分泌因子(ET,NTproBNP,hs-CRP)的影响. 上海市,上海市青浦区中医医院,2013-11-18.

[7]鲁文涛,程雪,吉红玉.参桂胶囊治疗慢性心力衰竭心肾阳虚患者临床观察[J].中西医结合心脑血管病杂志,2013,11(11):1306-1307.

[8]张春荣.参桂胶囊治疗终末期肾病伴心力衰竭临床研究[J].中医学报,2013,28(05):740-741.DOI:10.16368/j.issn.1674-8999.2013.05.033.

[9]俞春娟,王俊军,丁奇龙,钱玉萍. 参桂胶囊治疗慢性充血性心力衰竭疗效及对神经内分泌因子的影响[C]//.第 15 届中国南方国际心血管病学术会议专刊.,2013:239.

[10]俞春娟,王俊军,丁奇龙,钱玉萍,邢婷,和明丽.参桂胶囊治疗慢性充血性心力衰竭临床研究[J].中医学报,2013,28(04):570-572.DOI:10.16368/j.issn.1674-8999.2013.04.013.
[11]姚敏.参桂胶囊治疗慢性心力衰竭观察[J].医药论坛杂志,2010,31(12):88-89.
[12]桑凤梅.参桂胶囊结合西医常规治疗老年慢性心力衰竭 21 例疗效观察[J].中国医药导报,2010,7(16):84-85.
[13]耿秀双,李云富,党彦平.参桂胶囊辅助治疗重度充血性心力衰竭的临床疗效观察[J].中国药房,2008(30):2391-2392.
[14]殷惠军,蒋跃绒,刘颖,王承龙,郭艳.参桂胶囊对大鼠心肌梗死后心功能影响的研究[J].上海医药,2005(10):447-448.
[15]殷惠军,蒋跃绒,刘颖,王承龙,郭艳.参桂胶囊对大鼠心肌梗死后心功能影响的研究[J].中西医结合心脑血管病杂志,2004(08):466-467.
[16]殷惠军,蒋跃绒,刘颖,张颖.参桂胶囊对心肌梗死后心功能不全大鼠ET、Ang II 影响的研究[J].中西医结合心脑血管病杂志,2004(06):336-337.
[17]殷惠军,蒋跃绒,刘颖,张颖.参桂胶囊对心肌细胞能量代谢及脂质过氧化影响的研究[J].中医药信息,2004(03):71-72.

2. China Science and Technology Journal Database (VIP)

The database search in VIP was carried out on January 21, 2023, and a total of 15 literatures were found.

search strategy

M=(心力衰竭 OR 心衰 OR 心功能不全) AND M=参桂胶囊

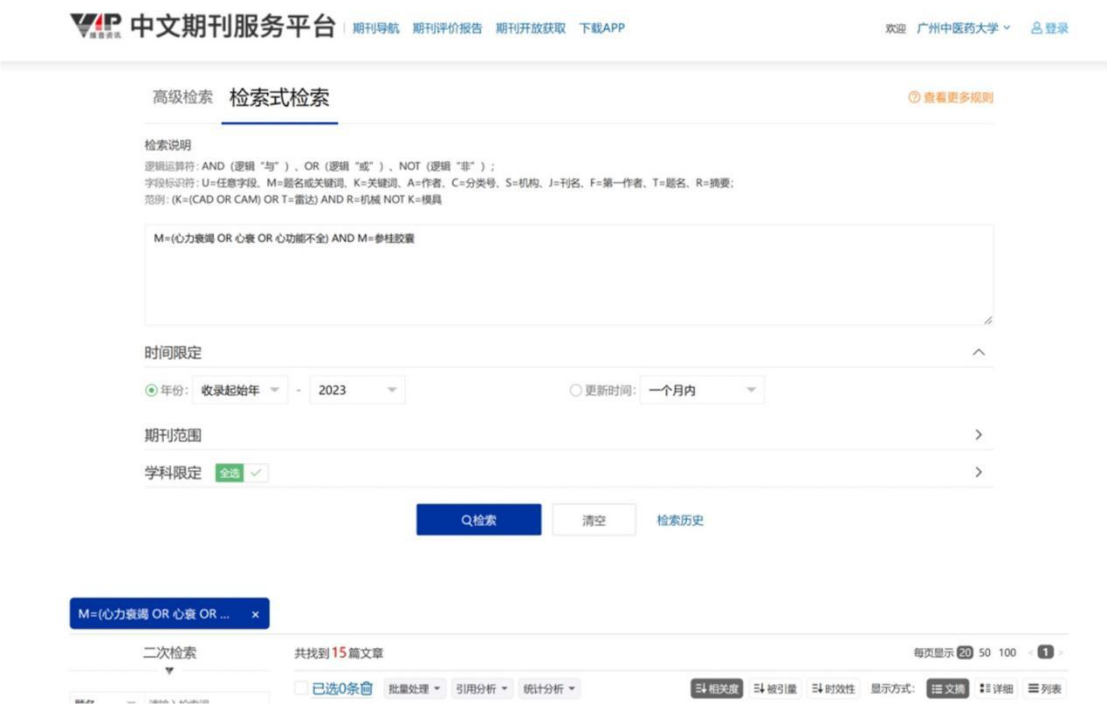

Supplementary Figure 2. Search strategy of VIP

[1]桑凤梅.参桂胶囊结合西医常规治疗老年慢性心力衰竭 21 例疗效观察[J].中国医药导报,2010,7(16):84-85.

[2]鲁文涛,程雪,吉红玉.参桂胶囊治疗慢性心力衰竭心肾阳虚患者临床观察[J].中西医结合心脑血管病杂志,2013,11(11):1306-1307.

[3]殷惠军,蒋跃绒,刘颖,张颖.参桂胶囊对心肌梗死后心功能不全大鼠 ET、Ang II 影响的研究[J].中西医结合心脑血管病杂志,2004,2(6):336-337.

[4]何潇言,严文萍.参桂胶囊治疗慢性心力衰竭的疗效及对 BNP 的影响研究[J].现代中西医结合杂志,2016,25(14):1512-1514.

[5]崔莹.参桂胶囊治疗慢性心力衰竭的临床研究[J].中西医结合心脑血管病杂志,2018,16(18):2670-2672.

[6]殷惠军,蒋跃绒,刘颖,王承龙,郭艳.参桂胶囊对大鼠心肌梗死后心功能影响的研究[J].中西医结合心脑血管病杂志,2004,2(8):466-467.

[7]殷惠军,蒋跃绒,刘颖,王承龙,郭艳.参桂胶囊对大鼠心肌梗死后心功能影响的研究[J].上海医药,2005,26(10):447-448.

[8]耿秀双,李云富,党彦平.参桂胶囊辅助治疗重度充血性心力衰竭的临床疗效观察[J].中国药房,2008,19(30):2391-2392.

[9]庄锐,吴旸,常佩芬,刘小芸,贺小芳,王尔玉,魏大为,张玲霞,朱海燕.参桂胶囊联合西药治疗冠心病慢性心力衰竭阳虚血瘀证患者临床疗效研究[J].辽宁中医药大学学报,2021,23(6):54-58.

[10]刘金锋.玉丹参桂胶囊治疗心力衰竭获国家发明专利[J].家庭用药,2012(12):41-41.
[11]张春荣.参桂胶囊治疗终末期肾病伴心力衰竭临床研究[J].中医学报,2013,28(5):740-741.
[12]俞春娟,王俊军,丁奇龙,钱玉萍,邢婷,和明丽.参桂胶囊治疗慢性充血性心力衰竭临床研究[J].中医学报,2013,28(4):570-572.
[13]姚敏.参桂胶囊治疗慢性心力衰竭观察[J].医药论坛杂志,2010(12):88-89.
[14]殷惠军,蒋跃绒,刘颖,张颖.参桂胶囊对心肌细胞能量代谢及脂质过氧化影响的研究[J].中医药信息,2004,21(3):71-72.
[15]刘金锋.玉丹参桂胶囊获上海医药行业名优产品称号[J].家庭用药,2013(1):68-68.

3. Wan Fang

The database search in Wan Fang was carried out on January 21, 2023, and a total of 21 literatures were found.

search strategy

主题:("心力衰竭" OR "心衰" OR "心功能不全") and 主题:("参桂胶囊")

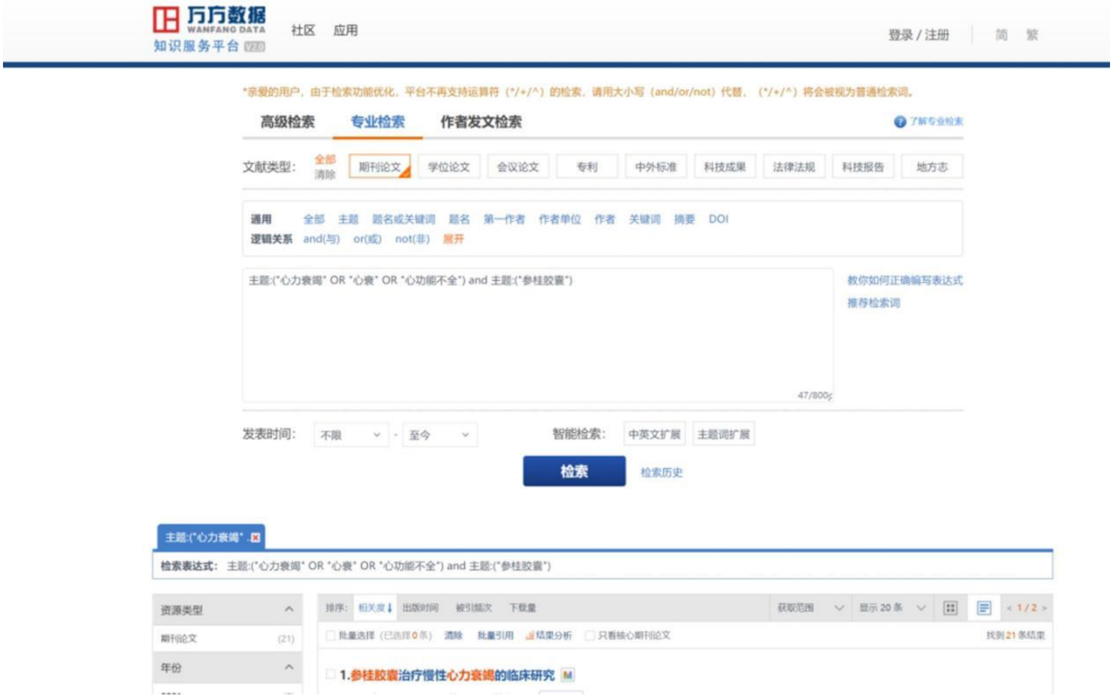

Supplementary Figure 3. Search strategy of Wan Fang

[1] 崔莹. 参桂胶囊治疗慢性心力衰竭的临床研究[J]. 中西医结合心脑血管病杂志,2018,16(18):2670-2672. DOI:10.12102/j.issn.1672-1349.2018.18.020.

[2] 肖晓,倪健俐,麻志恒. 从炎症及氧化应激角度观察真武汤加减对阳虚水泛型心力衰竭心肌重构的影响[J]. 中西医结合心脑血管病杂志,2021,19(2):293-296. DOI:10.12102/j.issn.1672-1349.2021.02.026.

[3] 何潇言,严文萍. 参桂胶囊治疗慢性心力衰竭的疗效及对 BNP 的影响研究[J]. 现代中西医结合杂志,2016,25(14):1512-1514. DOI:10.3969/j.issn.1008-8849.2016.14.010.

[4] 鲁文涛,程雪,吉红玉. 参桂胶囊治疗慢性心力衰竭心肾阳虚患者临床观察[J]. 中西医结合心脑血管病杂志,2013,11(11):1306-1307. DOI:10.3969/j.issn.1672-1349.2013.11.016.

[5] 桑凤梅. 参桂胶囊结合西医常规治疗老年慢性心力衰竭 21 例疗效观察[J]. 中国医药导报,2010,7(16):84-85. DOI:10.3969/j.issn.1673-7210.2010.16.044.

[6] 耿秀双,李云富,党彦平. 参桂胶囊辅助治疗重度充血性心力衰竭的临床疗效观察[J]. 中国药房,2008,19(30):2391-2392.

[7] 殷惠军,蒋跃绒,刘颖,等. 参桂胶囊对心肌梗死后心功能不全大鼠 ET、Ang II 影响的研究[J]. 中西医结合心脑血管病杂志,2004,2(6):336-337. DOI:10.3969/j.issn.1672-1349.2004.06.017.

[8] 陈群. 急诊内科老年重症心力衰竭临床治疗分析[J]. 中外女性健康研究,2015(11):161-162.

[9] 刘金锋. 玉丹参桂胶囊治疗心力衰竭获国家发明专利[J]. 家庭用药,2012(12):41.

[10] 姚敏. 参桂胶囊治疗慢性心力衰竭观察[J]. 医药论坛杂志,2010,31(12):88-89.

[11] 庄锐,吴旸,常佩芬,等. 参桂胶囊联合西药治疗冠心病慢性心力衰竭阳虚血瘀证患者临床疗效研究[J]. 辽宁中医药大学学报,2021,23(6):54-58. DOI:10.13194/j.issn.1673-842x.2021.06.013.
[12] 张昌生. 参桂胶囊辅助治疗老年慢性心力衰竭 26 例疗效观察[J]. 中外健康文摘,2012,9(22):276-277. DOI:10.3969/j.issn.1672-5085.2012.22.269.
[13] 殷惠军,蒋跃绒,刘颖,等. 参桂胶囊对大鼠心肌梗死后心功能影响的研究[J]. 中西医结合心脑血管病杂志,2004,2(8):466-467. DOI:10.3969/j.issn.1672-1349.2004.08.019.
[14] 殷惠军,蒋跃绒,刘颖,等. 参桂胶囊对大鼠心肌梗死后心功能影响的研究[J]. 上海医药,2005,26(10):447-448. DOI:10.3969/j.issn.1006-1533.2005.10.005.
[15] 殷惠军,蒋跃绒,刘颖,等. 参桂胶囊对心肌细胞能量代谢及脂质过氧化影响的研究[J]. 中医药信息,2004,21(3):71-72. DOI:10.3969/j.issn.1002-2406.2004.03.048.
[16] 刘博. 警惕冠心病引发心衰[J]. 家庭用药,2016(11):67.
[17] 许之民. 天气转冷谨防"心梗"和"心衰"[J]. 家庭用药,2017(11):23.
[18] 邓明华. 中西医结合治疗心肾综合征临床研究[J]. 现代医药卫生,2015(10):1537-1538. DOI:10.3969/j.issn.1009-5519.2015.10.038.
[19] 刘金锋. 玉丹参桂胶囊获上海医药行业名优产品称号[J]. 家庭用药,2013(1).
[20] 李锦祥. 益气温阳保心肾[J]. 家庭用药,2015(3):78.
[21] 达庆维. 中成药治疗糖尿病心脏并发症[J]. 家庭用药,2014(9):74.

4. China Biology Medicine disc (CBM)

The database search in CBM was carried out on January 21, 2023, and a total of 18 literatures were found.

search strategy

("参桂胶囊"[常用字段:智能]) AND ("心功能不全"[全部字段:智能] OR "心衰"[全部字段:智能] OR "心力衰竭"[全部字段:智能])

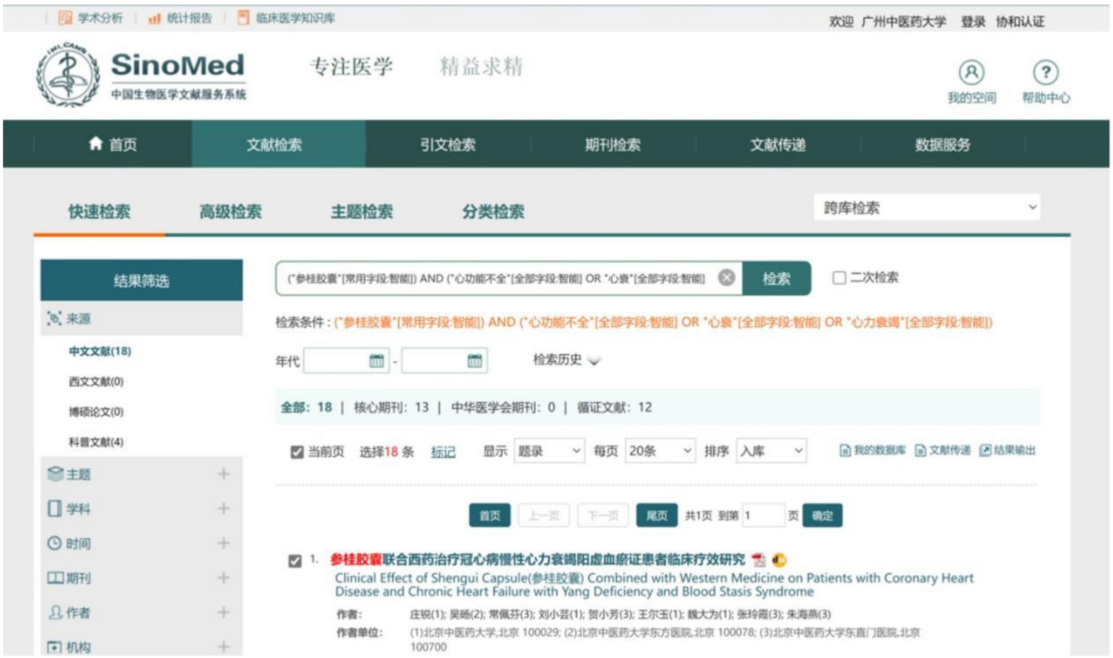

Supplementary Figure 4. Search strategy of CBM

[1] 庄锐,吴旸,常佩芬,等. 参桂胶囊联合西药治疗冠心病慢性心力衰竭阳虚血瘀证患者临床疗效研究[J]. 辽宁中医药大学学报,2021,23(6):54-58. DOI:10.13194/j.issn.1673-842x.2021.06.013.

[2] 肖晓,倪健俐,麻志恒. 从炎症及氧化应激角度观察真武汤加减对阳虚水泛型心力衰竭心肌重构的影响[J]. 中西医结合心脑血管病杂志,2021,19(2):293-296. DOI:10.12102/j.issn.1672-1349.2021.02.026.

[3] 崔莹. 参桂胶囊治疗慢性心力衰竭的临床研究[J]. 中西医结合心脑血管病杂志,2018,16(18):2670-2672. DOI:10.12102/j.issn.1672-1349.2018.18.020.

[4] 陈群. 急诊内科老年重症心力衰竭临床治疗分析[J]. 中外女性健康研究,2015(11):161-162.

[5] 何潇言,严文萍. 参桂胶囊治疗慢性心力衰竭的疗效及对 BNP 的影响研究[J]. 现代中西医结合杂志,2016,25(14):1512-1514. DOI:10.3969/j.issn.1008-8849.2016.14.010.

[6] 邓明华. 中西医结合治疗心肾综合征临床研究[J]. 现代医药卫生,2015(10):1537-1538. DOI:10.3969/j.issn.1009-5519.2015.10.038.

[7] 鲁文涛,程雪,吉红玉. 参桂胶囊治疗慢性心力衰竭心肾阳虚患者临床观察[J]. 中西医结合心脑血管病杂志,2013,11(11):1306-1307. DOI:10.3969/j.issn.1672-1349.2013.11.016.

[8] 李慧,杨守忠.玉丹参桂胶囊治疗慢性心力衰竭临床研究[J].中医学报,2013,28(07):1056-1057.DOI:10.16368/j.issn.1674-8999.2013.07.030.

[9] 张春荣.参桂胶囊治疗终末期肾病伴心力衰竭临床研究[J].中医学报,2013,28(5):740-741.
[10] 俞春娟,王俊军,丁奇龙,钱玉萍,邢婷,和明丽.参桂胶囊治疗慢性充血性心力衰竭临床研究[J].中医学报,2013,28(4):570-572.
[11] 张杰,张翥.玉丹参桂胶囊治疗心肾综合征临床研究[J].医药论坛杂志,2009,30(14):27-28.
[12] 姚敏.参桂胶囊治疗慢性心力衰竭观察[J].医药论坛杂志,2010(12):88-89.
[13] 桑凤梅. 参桂胶囊结合西医常规治疗老年慢性心力衰竭 21 例疗效观察[J]. 中国医药导报,2010,7(16):84-85. DOI:10.3969/j.issn.1673-7210.2010.16.044.
[14] 耿秀双,李云富,党彦平. 参桂胶囊辅助治疗重度充血性心力衰竭的临床疗效观察[J]. 中国药房,2008,19(30):2391-2392.
[15] 殷惠军,蒋跃绒,刘颖,等. 参桂胶囊对大鼠心肌梗死后心功能影响的研究[J]. 中西医结合心脑血管病杂志,2004,2(8):466-467. DOI:10.3969/j.issn.1672-1349.2004.08.019.
[16] 殷惠军,蒋跃绒,刘颖,等. 参桂胶囊对大鼠心肌梗死后心功能影响的研究[J]. 上海医药,2005,26(10):447-448. DOI:10.3969/j.issn.1006-1533.2005.10.005.
[17] 殷惠军,蒋跃绒,刘颖,等. 参桂胶囊对心肌细胞能量代谢及脂质过氧化影响的研究[J]. 中医药信息,2004,21(3):71-72. DOI:10.3969/j.issn.1002-2406.2004.03.048.
[18] 殷惠军,蒋跃绒,刘颖,等. 参桂胶囊对心肌梗死后心功能不全大鼠 ET、Ang II 影响的研究[J]. 中西医结合心脑血管病杂志,2004,2(6):336-337. DOI:10.3969/j.issn.1672-1349.2004.06.017.

5. PubMed

The database search in PubMed was carried out on January 21, 2023, and a total of 0 literature was found.

search strategy

Search number	Query	Search Details	Results	Time
3	(((((((((((((("Heart Failure"[Mesh]) OR (Cardiac Failure)) OR (Heart Decompensation)) OR (Decompensation, Heart)) OR (Heart Failure, Right-Sided)) OR (Heart Failure, Right Sided)) OR (Right-Sided Heart Failure)) OR (Right Sided Heart Failure)) OR (Myocardial Failure)) OR (Congestive Heart Failure)) OR (Heart Failure, Congestive)) OR (Heart Failure, Left-Sided)) OR (Heart Failure, Left Sided)) OR (Left-Sided Heart Failure)) OR (Left Sided Heart Failure)) AND (((Shengui capsule) OR (Shengui)) OR (Shen Gui capsule)) - Schema: all	("Heart Failure"[MeSH Terms] OR ("Cardiac"[All Fields] AND "Failure"[All Fields]) OR ("Heart"[All Fields] AND "Decompensation"[All Fields]) OR ("Decompensation"[All Fields] AND "Heart"[All Fields]) OR ("Heart"[All Fields] AND "Failure"[All Fields] AND "Right-Sided"[All Fields]) OR ("Heart"[All Fields] AND "Failure"[All Fields] AND "Right"[All Fields] AND "Sided"[All Fields]) OR ("Right-Sided"[All Fields] AND "Heart"[All Fields] AND "Failure"[All Fields]) OR ("Right"[All Fields] AND "Sided"[All Fields] AND "Heart"[All Fields] AND "Failure"[All Fields]) OR ("Myocardial"[All Fields] AND "Failure"[All Fields]) OR ("Congestive"[All Fields] AND "Heart"[All Fields] AND "Failure"[All Fields]) OR ("Heart"[All Fields] AND "Failure"[All Fields] AND "Congestive"[All Fields]) OR ("Heart"[All Fields] AND "Failure"[All Fields] AND "Left-Sided"[All Fields]) OR ("Heart"[All Fields] AND "Failure"[All Fields] AND "Left"[All Fields] AND "Sided"[All Fields]) OR ("Left-Sided"[All Fields] AND "Heart"[All Fields] AND "Failure"[All Fields]) OR ("Left"[All Fields] AND "Sided"[All Fields] AND "Heart"[All Fields] AND "Failure"[All Fields])) AND (("Shengui"[All Fields] AND "capsule"[All Fields]) OR "Shengui"[All Fields] OR ("Shen"[All Fields] AND "Gui"[All Fields] AND "capsule"[All Fields]))	0	21:31:51
2	((Shengui capsule) OR (Shengui)) OR (Shen Gui capsule)	("Shengui"[All Fields] AND ("capsule s"[All Fields] OR "capsules"[MeSH Terms] OR "capsules"[All Fields] OR "capsule"[All Fields])) OR "Shengui"[All Fields] OR (((shen, gui[Author] OR gui, shen[Author]) OR shen gui[Author] OR shen gui[Investigator]) AND ("capsule s"[All Fields] OR "capsules"[MeSH Terms] OR "capsules"[All Fields] OR "capsule"[All Fields]))	11	21:31:06
1	(((((((((((((("Heart Failure"[Mesh]) OR (Cardiac Failure)) OR (Heart Decompensation)) OR (Decompensation, Heart)) OR (Heart Failure, Right-Sided)) OR (Heart Failure, Right Sided)) OR (Right-Sided Heart Failure)) OR (Right Sided Heart Failure)) OR	"Heart Failure"[MeSH Terms] OR ("Heart Failure"[MeSH Terms] OR ("heart"[All Fields] AND "failure"[All Fields]) OR "Heart Failure"[All Fields] OR ("cardiac"[All Fields] AND "failure"[All Fields]) OR "cardiac failure"[All Fields]) OR ("Heart Failure"[MeSH Terms] OR ("heart"[All Fields] AND "failure"[All Fields]) OR "Heart Failure"[All Fields] OR ("heart"[All Fields] AND "decompensation"[All Fields]) OR "heart decompensation"[All Fields]) OR ("Heart Failure"[MeSH Terms]	333,685	21:29:49

(Myocardial Failure)) OR (Congestive Heart Failure)) OR (Heart Failure, Congestive)) OR (Heart Failure, Left-Sided)) OR (Heart Failure, Left Sided)) OR (Left-Sided Heart Failure)) OR (Left Sided Heart Failure)	OR ("heart"[All Fields] AND "failure"[All Fields]) OR "Heart Failure"[All Fields] OR ("decompensation"[All Fields] AND "heart"[All Fields]) OR "decompensation heart"[All Fields]) OR ("Heart Failure"[MeSH Terms] OR ("heart"[All Fields] AND "failure"[All Fields]) OR "Heart Failure"[All Fields] OR ("heart"[All Fields] AND "failure"[All Fields] AND "right"[All Fields] AND "sided"[All Fields]) OR "heart failure right sided"[All Fields]) OR ("Heart Failure"[MeSH Terms] OR ("heart"[All Fields] AND "failure"[All Fields]) OR "Heart Failure"[All Fields] OR ("heart"[All Fields] AND "failure"[All Fields] AND "right"[All Fields] AND "sided"[All Fields]) OR "heart failure right sided"[All Fields]) OR ("Heart Failure"[MeSH Terms] OR ("heart"[All Fields] AND "failure"[All Fields]) OR "Heart Failure"[All Fields] OR ("right"[All Fields] AND "sided"[All Fields] AND "heart"[All Fields] AND "failure"[All Fields]) OR "right sided heart failure"[All Fields]) OR ("Heart Failure"[MeSH Terms] OR ("heart"[All Fields] AND "failure"[All Fields]) OR "Heart Failure"[All Fields] OR ("right"[All Fields] AND "sided"[All Fields] AND "heart"[All Fields] AND "failure"[All Fields]) OR "right sided heart failure"[All Fields]) OR ("Heart Failure"[MeSH Terms] OR ("heart"[All Fields] AND "failure"[All Fields]) OR "Heart Failure"[All Fields] OR ("myocardial"[All Fields] AND "failure"[All Fields]) OR "myocardial failure"[All Fields]) OR ("Heart Failure"[MeSH Terms] OR ("heart"[All Fields] AND "failure"[All Fields]) OR "Heart Failure"[All Fields] OR ("congestive"[All Fields] AND "heart"[All Fields] AND "failure"[All Fields]) OR "congestive heart failure"[All Fields]) OR ("Heart Failure"[MeSH Terms] OR ("heart"[All Fields] AND "failure"[All Fields]) OR "Heart Failure"[All Fields] OR ("heart"[All Fields] AND "failure"[All Fields] AND "congestive"[All Fields]) OR "heart failure congestive"[All Fields]) OR ("Heart Failure"[MeSH Terms] OR ("heart"[All Fields] AND "failure"[All Fields]) OR "Heart Failure"[All Fields] OR ("heart"[All Fields] AND "failure"[All Fields] AND "left"[All Fields] AND "sided"[All Fields]) OR "heart failure left sided"[All Fields]) OR ("Heart Failure"[MeSH Terms] OR ("heart"[All Fields] AND "failure"[All Fields]) OR "Heart Failure"[All Fields] OR ("heart"[All Fields] AND "failure"[All Fields] AND "left"[All Fields] AND "sided"[All Fields]) OR "heart failure left sided"[All Fields]) OR ("Heart Failure"[MeSH Terms] OR ("heart"[All Fields] AND "failure"[All Fields]) OR "Heart Failure"[All Fields] OR ("left"[All Fields] AND "sided"[All Fields] AND "heart"[All Fields] AND "failure"[All Fields]) OR "left sided heart failure"[All Fields]) OR ("Heart Failure"[MeSH Terms] OR ("heart"[All

	Fields] AND "failure"[All Fields]) OR "Heart Failure"[All Fields] OR ("left"[All Fields] AND "sided"[All Fields] AND "heart"[All Fields] AND "failure"[All Fields]) OR "left sided heart failure"[All Fields])

An official website of the United States government Here's how you know

NIH National Library of Medicine
National Center for Biotechnology Information

Log in

PubMed Advanced Search Builder

PubMed.gov

User Guide

Add terms to the query box

All Fields | Enter a search term | ADD

Show Index

Query box

Enter / edit your search query here

Search

History and Search Details

Download | Delete

Search	Actions	Details	Query	Results	Time
#4	•••	>	Search: (((((((((((((("Heart Failure"[Mesh]) OR (Cardiac Failure)) OR (Heart Decompensation)) OR (Decompensation, Heart)) OR (Heart Failure, Right-Sided)) OR (Heart Failure, Right Sided)) OR (Right-Sided Heart Failure)) OR (Right Sided Heart Failure)) OR (Myocardial Failure)) OR (Congestive Heart Failure)) OR (Heart Failure, Congestive)) OR (Heart Failure, Left-Sided)) OR (Heart Failure, Left Sided)) OR (Left-Sided Heart Failure)) OR (Left Sided Heart Failure)) AND (((Shengui capsule) OR (Shengui)) OR (Shen Gui capsule))	0	21:39:21
#3	•••	>	Search: (((((((((((((("Heart Failure"[Mesh]) OR (Cardiac Failure)) OR (Heart Decompensation)) OR (Decompensation, Heart)) OR (Heart Failure, Right-Sided)) OR (Heart Failure, Right Sided)) OR (Right-Sided Heart Failure)) OR (Right Sided Heart Failure)) OR (Myocardial Failure)) OR (Congestive Heart Failure)) OR (Heart Failure, Congestive)) OR (Heart Failure, Left-Sided)) OR (Heart Failure, Left Sided)) OR (Left-Sided Heart Failure)) OR (Left Sided Heart Failure)) AND (((Shengui capsule) OR (Shengui)) OR (Shen Gui capsule)) - Schema: all	0	21:39:21
#2	•••	>	Search: ((Shengui capsule) OR (Shengui)) OR (Shen Gui capsule)	11	21:31:06
#1	•••	⌄	Search: ((((((((((((("Heart Failure"[Mesh]) OR (Cardiac Failure)) OR (Heart Decompensation)) OR (Decompensation, Heart)) OR (Heart Failure, Right-Sided)) OR (Heart Failure, Right Sided)) OR (Right-Sided Heart Failure)) OR (Right Sided Heart Failure)) OR (Myocardial Failure)) OR (Congestive Heart Failure)) OR (Heart Failure, Congestive)) OR (Heart Failure, Left-Sided)) OR (Heart Failure, Left Sided)) OR (Left-Sided Heart Failure)) OR (Left Sided Heart Failure)<br>"Heart Failure"[MeSH Terms] OR ("Heart Failure"[MeSH Terms] OR ("heart"[All Fields] AND "failure"[All Fields]) OR "Heart Failure"[All Fields] OR ("cardiac"[All Fields] AND "failure"[All Fields]) OR "cardiac failure"[All Fields]) OR ("Heart Failure"[MeSH Terms] OR ("heart"[All Fields] AND "failure"[All Fields]) OR "Heart Failure"[All Fields] OR ("heart"[All Fields] AND "decompensation"[All Fields]) OR "heart decompensation"[All Fields]) OR ("Heart Failure"[MeSH Terms] OR ("heart"[All Fields] AND "failure"[All Fields]) OR "Heart Failure"[All Fields] OR ("decompensation"[All Fields] AND "heart"[All Fields]) OR "decompensation heart"[All Fields]) OR ("Heart Failure"[MeSH Terms] OR ("heart"[All Fields] AND "failure"[All Fields]) OR "Heart Failure"[All Fields] OR ("heart"[All Fields] AND "failure"[All Fields] AND "right"[All Fields] AND "sided"[All Fields]) OR "heart failure right sided"[All Fields]) OR ("Heart Failure"[MeSH Terms] OR ("heart"[All Fields] AND "failure"[All Fields]) OR "Heart Failure"[All Fields] OR ("heart"[All Fields] AND "failure"[All Fields] AND "right"[All Fields] AND "sided"[All Fields]) OR "heart failure right sided"[All Fields]) OR ("Heart Failure"[MeSH Terms] OR ("heart"[All Fields] AND "failure"[All Fields]) OR "Heart Failure"[All Fields] OR ("right"[All Fields] AND "sided"[All Fields] AND "heart"[All Fields] AND "failure"[All Fields]) OR "right sided heart failure"[All Fields]) OR ("Heart Failure"[MeSH Terms] OR ("heart"[All Fields] AND "failure"[All Fields]) OR "Heart Failure"[All Fields] OR ("right"[All Fields] AND "sided"[All Fields] AND "heart"[All Fields] AND "failure"[All Fields]) OR "right sided heart failure"[All Fields]) OR ("Heart Failure"[MeSH Terms] OR ("heart"[All Fields] AND "failure"[All Fields]) OR "Heart Failure"[All Fields] OR ("myocardial"[All Fields] AND "failure"[All Fields]) OR "myocardial failure"[All Fields]) OR ("Heart Failure"[MeSH Terms] OR ("heart"[All Fields] AND "failure"[All Fields]) OR "Heart Failure"[All Fields] OR ("congestive"[All Fields] AND "heart"[All Fields] AND "failure"[All Fields]) OR "congestive heart failure"[All Fields]) OR ("Heart Failure"[MeSH Terms] OR ("heart"[All Fields] AND "failure"[All Fields]) OR "Heart Failure"[All Fields] OR ("heart"[All Fields] AND "failure"[All Fields] AND "congestive"[All Fields]) OR "heart failure congestive"[All Fields]) OR ("Heart Failure"[MeSH Terms] OR ("heart"[All Fields] AND "failure"[All Fields]) OR "Heart Failure"[All Fields] OR ("heart"[All Fields] AND "failure"[All Fields] AND "left"[All Fields] AND "sided"[All Fields]) OR "heart failure left sided"[All Fields]) OR ("Heart Failure"[MeSH Terms] OR ("heart"[All Fields] AND "failure"[All Fields]) OR "Heart Failure"[All Fields] OR ("heart"[All Fields] AND "failure"[All Fields] AND "left"[All Fields] AND "sided"[All Fields]) OR "heart failure left sided"[All Fields]) OR ("Heart Failure"[MeSH Terms] OR ("heart"[All Fields] AND "failure"[All Fields]) OR "Heart Failure"[All Fields] OR ("left"[All Fields] AND "sided"[All Fields] AND "heart"[All Fields] AND "failure"[All Fields]) OR "left sided heart failure"[All Fields]) OR ("Heart Failure"[MeSH Terms] OR ("heart"[All Fields] AND "failure"[All Fields]) OR "Heart Failure"[All Fields] OR ("left"[All Fields] AND "sided"[All Fields] AND "heart"[All Fields] AND "failure"[All Fields]) OR "left sided heart failure"[All Fields])	333,685	21:29:49

Supplementary Figure 5. Search strategy of Pubmed

6. Embase

The database search in Embase was carried out on January 21, 2023, and a total of 0 literature was found.

search strategy

No.	Query	Results	Date
#3	#1 AND #2	0	?-January-23
#2	'shengui' OR 'shengui capsule'	25	?-January-23
#1	'heart failure'/exp OR 'heart failure' OR 'cardiac failure' OR 'heart decompensation' OR 'decompensation, heart' OR 'heart failure, right-sided' OR 'heart failure, right sided' OR 'right-sided heart failure' OR 'right sided heart failure' OR 'myocardial failure' OR 'congestive heart failure' OR 'heart failure, congestive' OR 'heart failure, left-sided' OR 'heart failure, left sided' OR 'left-sided heart failure' OR 'left sided heart failure'	701,639	?-January-23

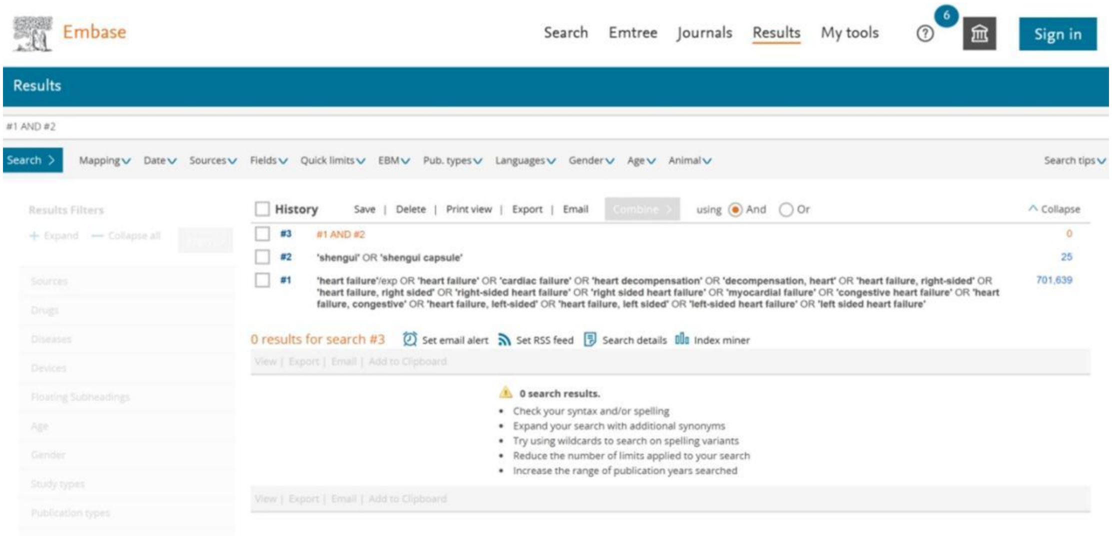

Supplementary Figure 6. Search strategy of Embase

7. Cochrane Library

The database search in Cochrane Library was carried out on January 21, 2023, and a total of 0 literature was found.

search strategy

ID Search

#1 (Heart Failure):ti,ab,kw OR (Cardiac Failure):ti,ab,kw OR (Heart Decompensation):ti,ab,kw OR (Decompensation, Heart):ti,ab,kw OR (Heart Failure, Right-Sided):ti,ab,kw OR (Heart Failure, Right Sided):ti,ab,kw OR (Right-Sided Heart Failure):ti,ab,kw OR (Right Sided Heart Failure):ti,ab,kw OR (Myocardial Failure):ti,ab,kw OR (Congestive Heart Failure):ti,ab,kw OR (Heart Failure, Congestive):ti,ab,kw OR (Heart Failure, Left-Sided):ti,ab,kw OR (Heart Failure, Left Sided):ti,ab,kw OR (Left-Sided Heart Failure):ti,ab,kw OR (Left Sided Heart Failure):ti,ab,kw(Word variations have been searched)

#2 (Shengui capsule):ti,ab,kw OR (Shengui):ti,ab,kw OR (Shen Gui capsule):ti,ab,kw(Word variations have been searched)

#3 #1 and #2

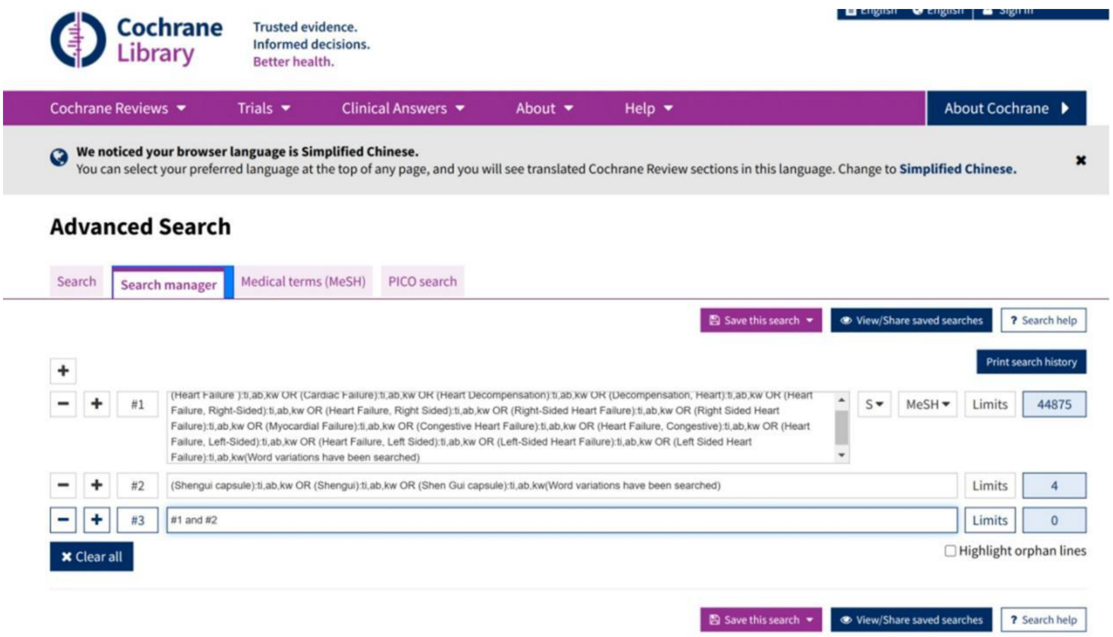

Supplementary Figure 7. Search strategy of Cochrane Library

Supplementary Material S7. Literature screening process

1. List of studies searched after removing duplicate studies

[1]李松林,谷朝华,马涛,姚先丽.参桂胶囊联合比索洛尔治疗慢性心力衰竭的临床研究[J/OL].现代药物与临床:1-4.http://kns.cnki.net/kcms/detail/12.1407.R.20221228.2204.006.html

[2]庄锐,吴旸,常佩芬,刘小芸,贺小芳,王尔玉,魏大为,张玲霞,朱海燕.参桂胶囊联合西药治疗冠心病慢性心力衰竭阳虚血瘀证患者临床疗效研究[J].辽宁中医药大学学报,2021,23(06):54-58.DOI:10.13194/j.issn.1673-842x.2021.06.013.

[3]崔莹.参桂胶囊治疗慢性心力衰竭的临床研究[J].中西医结合心脑血管病杂志,2018,16(18):2670-2672.

[4]何潇言,严文萍.参桂胶囊治疗慢性心力衰竭的疗效及对BNP的影响研究[J].现代中西医结合杂志,2016,25(14):1512-1514.

[5]俞春娟, 参桂胶囊在慢性充血性心力衰竭中的应用及对神经内分泌因子(ET,NTproBNP,hs-CRP)的影响. 上海市,上海市青浦区中医医院,2013-11-18.

[6]鲁文涛,程雪,吉红玉.参桂胶囊治疗慢性心力衰竭心肾阳虚患者临床观察[J].中西医结合心脑血管病杂志,2013,11(11):1306-1307.

[7]张春荣.参桂胶囊治疗终末期肾病伴心力衰竭临床研究[J].中医学报,2013,28(05):740-741.DOI:10.16368/j.issn.1674-8999.2013.05.033.

[8]俞春娟,王俊军,丁奇龙,钱玉萍. 参桂胶囊治疗慢性充血性心力衰竭疗效及对神经内分泌因子的影响[C]//.第 15 届中国南方国际心血管病学术会议专刊.,2013:239.

[9]俞春娟,王俊军,丁奇龙,钱玉萍,邢婷,和明丽.参桂胶囊治疗慢性充血性心力衰竭临床研究[J].中医学报,2013,28(04):570-572.DOI:10.16368/j.issn.1674-8999.2013.04.013.

[10]姚敏.参桂胶囊治疗慢性心力衰竭观察[J].医药论坛杂志,2010,31(12):88-89.

[11]桑凤梅.参桂胶囊结合西医常规治疗老年慢性心力衰竭 21 例疗效观察[J].中国医药导报,2010,7(16):84-85.

[12]耿秀双,李云富,党彦平.参桂胶囊辅助治疗重度充血性心力衰竭的临床疗效观察[J].中国药房,2008(30):2391-2392.

[13]殷惠军,蒋跃绒,刘颖,王承龙,郭艳.参桂胶囊对大鼠心肌梗死后心功能影响的研究[J].上海医药,2005(10):447-448.

[14]殷惠军,蒋跃绒,刘颖,王承龙,郭艳.参桂胶囊对大鼠心肌梗死后心功能影响的研究[J].中西医结合心脑血管病杂志,2004(08):466-467.

[15]殷惠军,蒋跃绒,刘颖,张颖.参桂胶囊对心肌梗死后心功能不全大鼠ET、Ang II 影响的研究[J].中西医结合心脑血管病杂志,2004(06):336-337.

[16]殷惠军,蒋跃绒,刘颖,张颖.参桂胶囊对心肌细胞能量代谢及脂质过氧化影响的研究[J].中医药信息,2004(03):71-72.

[17]肖晓,倪健俐,麻志恒. 从炎症及氧化应激角度观察真武汤加减对阳虚水泛型心力衰竭心肌重构的影响[J]. 中西医结合心脑血管病杂志,2021,19(2):293-296. DOI:10.12102/j.issn.1672-1349.2021.02.026.

[18]陈群. 急诊内科老年重症心力衰竭临床治疗分析[J]. 中外女性健康研究,2015(11):161-16

[19]刘金锋. 玉丹参桂胶囊治疗心力衰竭获国家发明专利[J]. 家庭用药,2012(12):41.

[20]张昌生. 参桂胶囊辅助治疗老年慢性心力衰竭 26 例疗效观察[J]. 中外健康文摘,2012,9(22):276-277. DOI:10.3969/j.issn.1672-5085.2012.22.269.

[21] 刘博. 警惕冠心病引发心衰[J]. 家庭用药,2016(11):67.
[22] 许之民. 天气转冷谨防“心梗”和“心衰”[J]. 家庭用药,2017(11):23.
[23] 邓明华. 中西医结合治疗心肾综合征临床研究[J]. 现代医药卫生,2015(10):1537-1538. DOI:10.3969/j.issn.1009-5519.2015.10.038.
[24] 刘金锋. 玉丹参桂胶囊获上海医药行业名优产品称号[J]. 家庭用药,2013(1).
[25] 李锦祥. 益气温阳保心肾[J]. 家庭用药,2015(3):78.
[26] 达庆维. 中成药治疗糖尿病心脏并发症[J]. 家庭用药,2014(9):74.
[27] 李慧,杨守忠.玉丹参桂胶囊治疗慢性心力衰竭临床研究[J].中医学报,2013,28(07):1056-1057.DOI:10.16368/j.issn.1674-8999.2013.07.030.
[28] 张杰,张翥.玉丹参桂胶囊治疗心肾综合征临床研究[J].医药论坛杂志,2009,30(14):27-28.

2. List of studies excluded after reading title and abstract

[1] 俞春娟, 参桂胶囊在慢性充血性心力衰竭中的应用及对神经内分泌因子(ET,NTproBNP,hs-CRP)的影响. 上海市,上海市青浦区中医医院,2013-11-18.（非RCT）
[2] 张春荣.参桂胶囊治疗终末期肾病伴心力衰竭临床研究[J].中医学报,2013,28(05):740-741. DOI:10.16368/j.issn.1674-8999.2013.05.033.（非慢性心衰）
[3] 殷惠军,蒋跃绒,刘颖,王承龙,郭艳.参桂胶囊对大鼠心肌梗死后心功能影响的研究[J].中西医结合心脑血管病杂志,2004(08):466-467.（非临床试验）
[4] 殷惠军,蒋跃绒,刘颖,张颖.参桂胶囊对心肌梗死后心功能不全大鼠ET、Ang II 影响的研究[J].中西医结合心脑血管病杂志,2004(06):336-337.（非临床试验）
[5] 殷惠军,蒋跃绒,刘颖,张颖.参桂胶囊对心肌细胞能量代谢及脂质过氧化影响的研究[J].中医药信息,2004(03):71-72.（非临床试验）
[6] 殷惠军,蒋跃绒,刘颖,王承龙,郭艳.参桂胶囊对大鼠心肌梗死后心功能影响的研究[J].上海医药,2005(10):447-448.（非临床试验）
[7] 肖晓,倪健俐,麻志恒. 从炎症及氧化应激角度观察真武汤加减对阳虚水泛型心力衰竭心肌重构的影响[J]. 中西医结合心脑血管病杂志,2021,19(2):293-296. DOI:10.12102/j.issn.1672-1349.2021.02.026.（非参桂胶囊）
[8] 刘金锋. 玉丹参桂胶囊治疗心力衰竭获国家发明专利[J]. 家庭用药,2012(12):41.（非参桂胶囊）
[9] 刘博. 警惕冠心病引发心衰[J]. 家庭用药,2016(11):67.（非RCT）
[10] 许之民. 天气转冷谨防“心梗”和“心衰”[J]. 家庭用药,2017(11):23.（非RCT）
[11] 邓明华. 中西医结合治疗心肾综合征临床研究[J]. 现代医药卫生,2015(10):1537-1538. DOI:10.3969/j.issn.1009-5519.2015.10.038.（非慢性心衰）
[12] 刘金锋. 玉丹参桂胶囊获上海医药行业名优产品称号[J]. 家庭用药,2013(1).（非RCT）
[13] 李锦祥. 益气温阳保心肾[J]. 家庭用药,2015(3):78.（非RCT）
[14] 达庆维. 中成药治疗糖尿病心脏并发症[J]. 家庭用药,2014(9):74.（非RCT）
[15] 李慧,杨守忠.玉丹参桂胶囊治疗慢性心力衰竭临床研究[J].中医学报,2013,28(07):1056-1057.DOI:10.16368/j.issn.1674-8999.2013.07.030.（非参桂胶囊）
[16] 张杰,张翥.玉丹参桂胶囊治疗心肾综合征临床研究[J].医药论坛杂志,2009,30(14):27-28.（非参桂胶囊）

3 List of studies excluded after reading full-text and reasons

[1] 俞春娟,王俊军,丁奇龙,钱玉萍. 参桂胶囊治疗慢性充血性心力衰竭疗效及对神经内分泌因子的影响[C]//.第 15 届中国南方国际心血管病学术会议专刊.,2013:239.（会议文献，无研究的详细内容缺失）
[2] 张昌生. 参桂胶囊辅助治疗老年慢性心力衰竭 26 例疗效观察[J]. 中外健康文摘,2012,9(22):276-277. DOI:10.3969/j.issn.1672-5085.2012.22.269.（非 RCT，回顾性研究）
[3] 姚敏.参桂胶囊治疗慢性心力衰竭观察[J].医药论坛杂志,2010,31(12):88-89.（非 RCT，未提及随机分组）

4. Total studies includes in reviews

[1] 李松林,谷朝华,马涛,姚先丽.参桂胶囊联合比索洛尔治疗慢性心力衰竭的临床研究[J/OL].现代药物与临床:1-4.http://kns.cnki.net/kcms/detail/12.1407.R.20221228.2204.006.html
[2] 庄锐,吴旸,常佩芬,刘小芸,贺小芳,王尔玉,魏大为,张玲霞,朱海燕.参桂胶囊联合西药治疗冠心病慢性心力衰竭阳虚血瘀证患者临床疗效研究[J].辽宁中医药大学学报,2021,23(06):54-58.DOI:10.13194/j.issn.1673-842x.2021.06.013.
[3] 崔莹.参桂胶囊治疗慢性心力衰竭的临床研究[J].中西医结合心脑血管病杂志,2018,16(18):2670-2672.
[4]何潇言,严文萍.参桂胶囊治疗慢性心力衰竭的疗效及对 BNP 的影响研究[J].现代中西医结合杂志,2016,25(14):1512-1514.
[5]鲁文涛,程雪,吉红玉.参桂胶囊治疗慢性心力衰竭心肾阳虚患者临床观察[J].中西医结合心脑血管病杂志,2013,11(11):1306-1307.
[6]俞春娟,王俊军,丁奇龙,钱玉萍,邢婷,和明丽.参桂胶囊治疗慢性充血性心力衰竭临床研究[J].中医学报,2013,28(04):570-572.DOI:10.16368/j.issn.1674-8999.2013.04.013.
[7]桑凤梅.参桂胶囊结合西医常规治疗老年慢性心力衰竭 21 例疗效观察[J].中国医药导报,2010,7(16):84-85.
[8]耿秀双,李云富,党彦平.参桂胶囊辅助治疗重度充血性心力衰竭的临床疗效观察[J].中国药房,2008(30):2391-2392.
[9]陈群. 急诊内科老年重症心力衰竭临床治疗分析[J]. 中外女性健康研究,2015(11):161-16

Supplementary File S8. Results of subgroup analysis

6.1 Subgroup analysis of the LVEF according to the SGCP does.

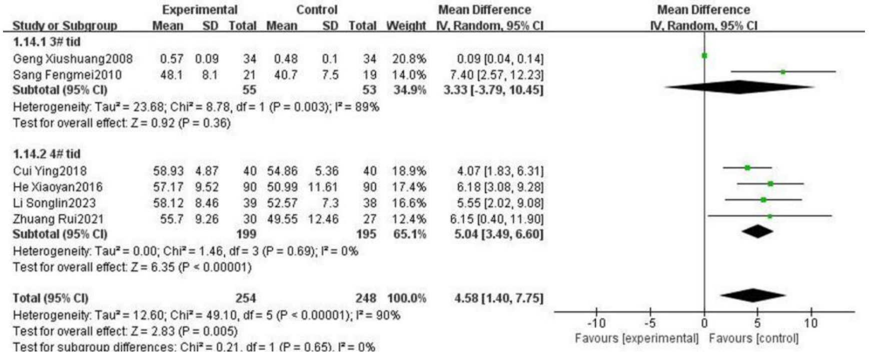

Supplementary Figure 8. Subgroup analysis of the LVEF according to the SGCP does.

6.2 Subgroup analysis of the LVEF according to the age.

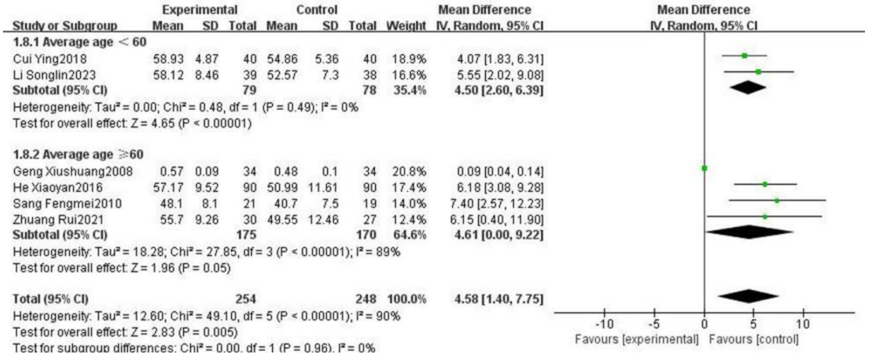

Supplementary Figure 9. Subgroup analysis of the LVEF according to the age.

6.3 Subgroup analysis of the effective rate according to the SGCP does.

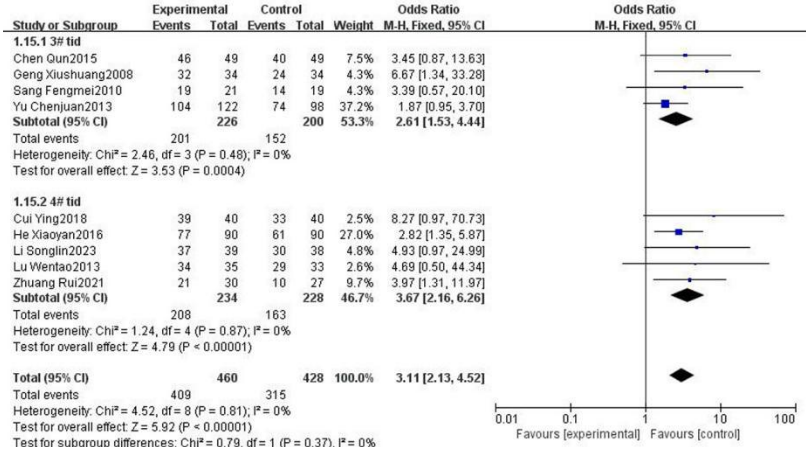

Supplementary Figure 10. Subgroup analysis of the effective rate according to the SGCP does.

6.4 Subgroup analysis of the effective rate according to the age.

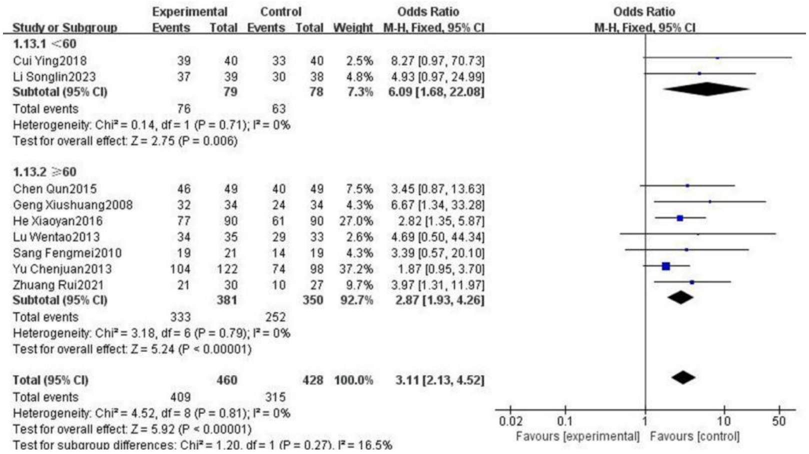

Supplementary Figure 11. Subgroup analysis of the effective rate according to the age.

6.5 Subgroup analysis of BNP according to the age.

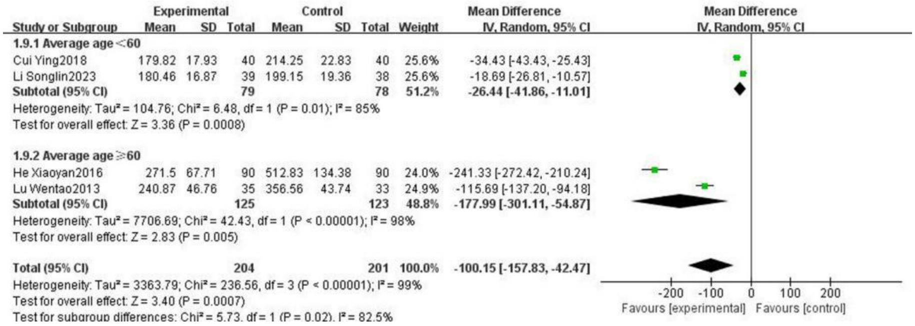

Supplementary Figure 12. Subgroup analysis of BNP according to the age.
